# Variants at the MHC Region Associate With Susceptibility to *Clostridioides difficile* Infection: A Genome-Wide Association Study Using Comprehensive Electronic Health Records

**DOI:** 10.3389/fimmu.2021.638913

**Published:** 2021-03-25

**Authors:** Jiang Li, Yanfei Zhang, Alexandria L. Jilg, Donna M. Wolk, Harshit S. Khara, Amy Kolinovsky, David D. K. Rolston, Raquel Hontecillas, Josep Bassaganya-Riera, Marc S. Williams, Vida Abedi, Ming Ta Michael Lee

**Affiliations:** ^1^ Department of Molecular and Functional Genomics, Geisinger, Danville, PA, United States; ^2^ Genomic Medicine Institute, Geisinger, Danville, PA, United States; ^3^ Department of Internal Medicine, Geisinger, Danville, PA, United States; ^4^ Diagnostic Medicine Institute, Department of Laboratory Medicine, Geisinger, Danville, PA, United States; ^5^ Department of Gastroenterology and Hepatology, Geisinger, Danville, PA, United States; ^6^ Phenotype Core, Geisinger, Danville, PA, United States; ^7^ The NIMML Institute, Blacksburg, VA, United States; ^8^ Regeneron Genetics Center, Tarrytown, NY, United States

**Keywords:** *Clostridioides difficile*, MICA, C4a, NOTCH4, GWAS

## Abstract

**Background:**

*Clostridioides difficile* is a major cause of healthcare-associated and community-acquired diarrhea. Host genetic susceptibility to *Clostridioides difficile* infection has not been studied on a large-scale.

**Methods:**

A total of 1,160 *Clostridioides difficile* infection cases and 15,304 controls were identified by applying the eMERGE *Clostridioides difficile* infection algorithm to electronic health record data. A genome-wide association study was performed using a linear mixed model, adjusted for significant covariates in the full dataset and the antibiotic subgroup. Colocalization and MetaXcan were performed to identify potential target genes in *Clostridioides difficile* infection - relevant tissue types.

**Results:**

No significant genome-wide association was found in the meta-analyses of the full *Clostridioides difficile* infection dataset. One genome-wide significant variant, rs114751021, was identified (OR = 2.42; 95%CI = 1.84-3.11; p=4.50 x 10^-8^) at the major histocompatibility complex region associated with *Clostridioides difficile* infection in the antibiotic group. Colocalization and MetaXcan identified *MICA*, *C4A/C4B*, and *NOTCH4* as potential target genes. Down-regulation of *MICA*, upregulation of *C4A* and *NOTCH4* was associated with a higher risk for *Clostridioides difficile* infection.

**Conclusions:**

Leveraging the EHR and genetic data, genome-wide association, and fine-mapping techniques, this study identified variants and genes associated with *Clostridioides difficile* infection, provided insights into host immune mechanisms, and described the potential for novel treatment strategies for *Clostridioides difficile* infection. Future replication and functional validation are needed.

## Introduction

*Clostridioides difficile* (*C. difficile*) is an anaerobic, Gram-positive, and spore-forming bacterium. *C. difficile* is a major cause of antibiotic-associated diarrhea. It is also associated with the community-acquisition of diarrhea. A surveillance study across 10 geographic areas in the United States in 2011 estimated 453,000 incident *C. difficile* infections (CDI) and 29,000 associated deaths per year (1). Individuals who harbor *C. difficile* in their gut may be carriers with no discernible symptoms, may have diarrhea of variable severity, or may progress from fulminant colitis to systemic disease and death (2). The clinical manifestations are determined by several major factors, including the strain type and associated virulence (3), the host immune response (4), disruptions in the host’s microbiome (for example, those caused by antibiotics) (5), patient medications (6), and gastric acid suppression (7).

Toxins are the major virulence factors of *C. difficile* (8). Toxins A (TcdA) and B (TcdB) are large secreted glucosyltransferase proteins that target intestinal epithelial cells and disrupt the epithelial barrier leading to secretory diarrhea (9). The hypervirulent BI/NAP1/027 strain is often fluoroquinolone resistant and the most common cause of CDI globally (10, 11). Host risk factors include advanced age (≥ 65 years), underlying comorbidities such as inflammatory bowel disease (IBD), organ transplantation, immunodeficiency, certain medications including antibiotics, chemotherapy, and proton pump inhibitor (PPI) (7), and prolonged hospital stay (4). Antibiotic use and altered intestinal microbiota can reduce colonization resistance against pathogens, including *C. difficile*, therefore, certain antimicrobial agents remain the most prominent risk factors for CDI (4, 12).

Multiple studies have investigated the genetics of the bacteria and identified genes and genetic variation that are associated with the virulence factors, deepening our understanding of the emergence and global spread of *C. difficile* strains (11, 13, 14). However, the identification of host genetic susceptibility to CDI has not been extensively investigated. Several candidate gene studies have mainly focused on proinflammatory cytokine genes such as interleukin-8 (*IL-8)* (15–17), or IBD-associated single nucleotide variants (SNVs) (18, 19). One SNV, rs4073(–251T>A), found in the promoter region of *IL8*, was reported to be associated with CDI (15) and recurrent CDI (16). However, this association was not validated in independent studies (17). Eight IBD-associated SNVs were found associated with CDI at a nominal significance level (p<0.05) in an ulcerative colitis population (18). Another study reported that rs2243250, an *IL4*-associated SNV, was associated with CDI in an IBD population (19). Hitherto, only one study with 57 CDI patients performed a genome-wide association study (GWAS) in a cohort of patients with multiple myeloma undergoing autologous stem cell transplantation. None of these associations reached genome-wide significance (20).

All the published studies suffer from small sample size (CDI cases < 60) that limit the statistical power. There is an unmet need to identify host genetic risk factors for CDI in general and in high-risk populations such as antibiotics-users. In this study, we leveraged the electronic health record (EHR) linked to genetic data and performed GWAS to identify common variants associated with CDI.

## Methods

### Study Cohort

**Figure 1:**
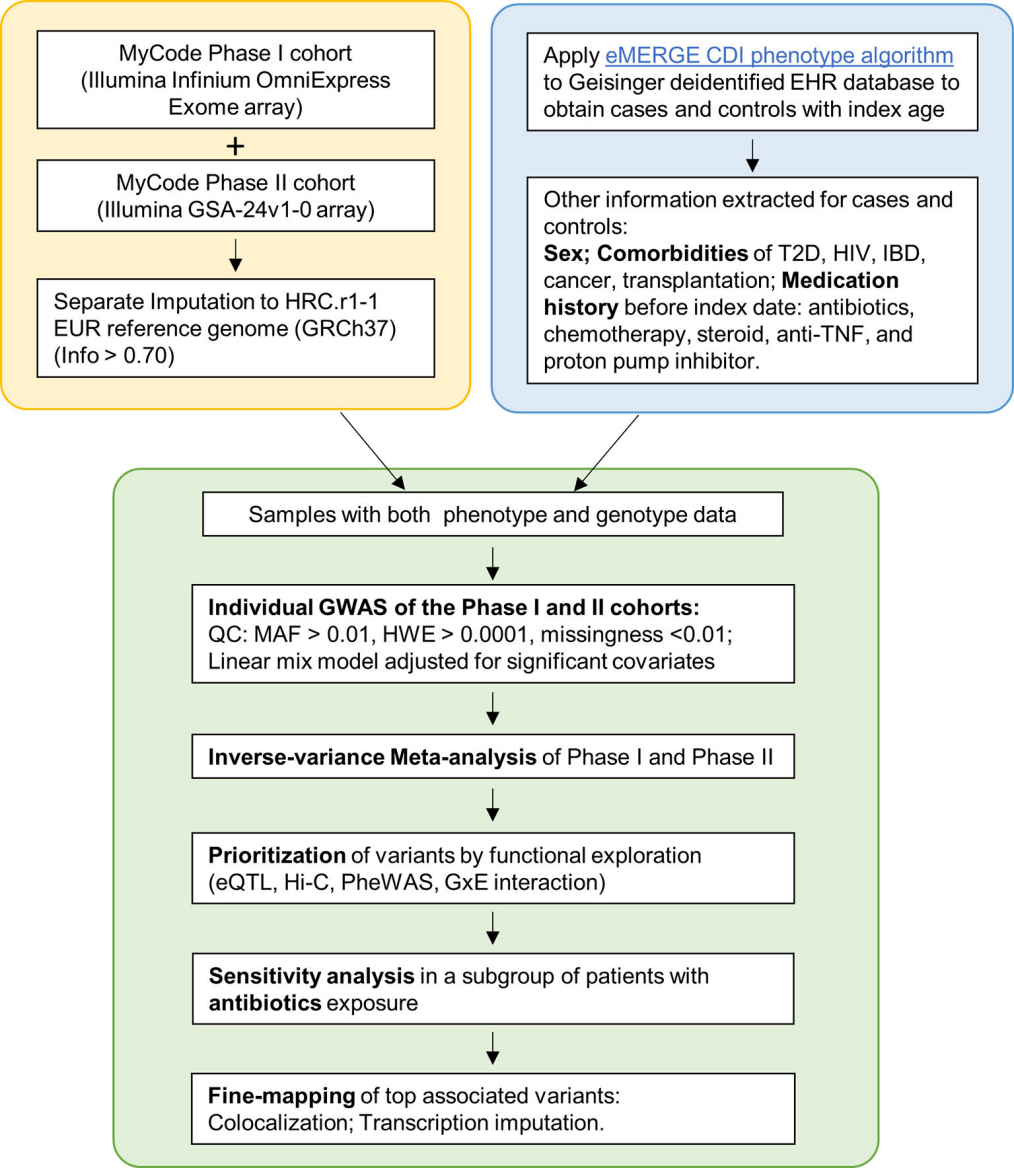
Flowchart of the study. Yellow box: Genotyping and imputation was conducted separately for Phase I and II. Blue box: eMERGE CDI algorithm was applied to identify cases and controls. Clinical information was extracted. Green box: individual GWAS and meta-analyses, followed by a sensitivity analysis and Fine-mapping.

The MyCode^®^ Community Health Initiative (MyCode) is an ongoing project with deidentified EHR linked to genomic data supporting genetic studies (21–23). The primary cohort in this study consist of approximately 92,000 consented participants from MyCode Phase I (approximately 60,000 participants) and Phase II (approximately 32,000 participants). This study was exempted as non-human subjects research by the Geisinger Institutional Review Board for using de-identified information and was approved by the MyCode Governing Board. **Figure 1** illustrates the study design and pipeline for sample selection and data analysis.

### Definition of CDI Cases and Controls and Extraction of Clinical Variables

Geisinger houses a continually growing deidentified EHR database for research, including information describing patients’ demographics, diagnosis, laboratory test results, prescriptions, procedures, and vital signs. A phenotyping algorithm to identify CDI cases and controls from EHR data was developed by the eMERGE Network and was implemented in the Geisinger EHR (dbGaP study accession: phs000888.v1.p1; phenotype accession: phd004976.1). Briefly, the eMERGE algorithm identified CDI cases using the reference standard, based on positive laboratory results, or the silver standard, based on diagnosis codes combined with information from the patients’ chart. Controls were defined as patients without CDI, based on negative laboratory results, but exposed to similar risk factors, such as antibiotic use, hospitalization, high-risk conditions, and medications (details in the **Supplementary Note**). Polymerase chain reaction (PCR) was used as the laboratory reference standard test with the BD GeneOhm™ Cdiff Assay (Becton Dickinson, Franklin Lakes, NJ) used prior to mid-April 2014 and the Xpert *C. difficile*/Epi assay (Cepheid, Sunnyvale CA) used after that. Only adults (age >=18) were included in this study. Risk factors and demographic information were extracted from EHR data. Using data through Dec 31, 2018, we identified 946 cases and 10,840 controls from Phase I and 214 cases and 3,304 controls from Phase II. Of these, 587 cases and 3,166 controls had antibiotics exposure prior to the index date.

### Genetic Association Tests

The genotyping, imputation and quality control for the genetic data have been previously described (24) (**Figure 1**; **Supplementary Methods**). Pairs of individuals with first- or second-degree relatedness were identified (**Supplementary Figure 1A**). Principal component analyses indicated that the individuals in this study are of European ancestry (**Supplementary Figure 1B**). In total, 7,077,672 SNVs from Phase I, 6,683,047 SNVs from Phase II, and 6,497,3696 SNVs from the merged antibiotic-exposed group were included in the analyses.

BOLT-LMM, a linear mixed model, was adopted to test genetic associations while accounting for covariates and cryptic relatedness between individuals (25). In the sensitivity analysis of antibiotics subgroups, only the patients exposed to antibiotic risk within 7 to 62 days before the index date, were included. The significant covariates between cases and controls were adjusted. The fixed effect, inverse variance weighted meta-analyses of Phase I and II summary statistics were accomplished using METAL (26). SAIGE, which uses the saddle point approximation to calibrate the distribution of score test statistics, was also applied to account for case-control imbalance (27). GEMMA was used to test the gene x environment (GxE) interaction, which employs a linear mixed model to control for the main effects of SNV and environmental factors while testing for the interaction effect (28). The odds ratio (OR) was converted from the regression coefficient β in R (29). Open Targets Genetics was queried for the top associated SNVs to evaluate whether other significantly associated phenotypes were present.

### Colocalization and Transcriptome-Imputation Based Gene-Level Association

Statistical fine-mapping using functional genomics data, such as expression quantitative trait loci (eQTL), has been used to explore the potential mechanisms of GWAS variants on diseases. Colocalization is one commonly used method that integrates GWAS and eQTL data to estimate the probability of the same variant being causal in both GWAS and eQTL studies (30). Colocalized signals provide evidence of possible causal relationships between the target gene of an eQTL and the GWAS trait. To identify causal genes and target tissues, we performed colocalization analyses of the four top associated variants at the MHC region with CDI in the antibiotic subgroup using eCAVIAR (31). The eQTL data of the whole blood and the seven gastrointestinal (GI) tissue types (colon sigmoid, colon transverse, esophageal mucosa, esophageal muscularis, gastroesophageal junction, small intestine, and stomach) from GTEx v7 data release (32) were included in the analyses. The overlapped SNVs in the GWAS and GTEx were included in the analyses. Fifty SNVs, upstream and downstream from the GWAS lead SNV in each locus, were included to calculate the colocalization posterior probability (CLPP) assuming a maximum of two causal SNVs in each locus. Only significant eGenes were examined. All SNV-gene pairs eQTL data for each eGene were then used in the analyses. CLPP represents the probability that the same variant is causal in both GWAS and QTLs. We adopted a CLPP > 0.01 as the colocalization cutoff (31). Variants that have CLPP > 0.01 and GWAS p-value <10^-5^ were included to identify target genes. We report the highest probability (max of CLPP value) for each target gene in the relevant tissue.

### S-PrediXcan Transcriptome-Imputation Based Gene-Level Association

S-PrediXcan is an integrative gene-based association approach that uses summary data and pre-imputed transcriptome levels with models trained in a measured transcriptome dataset (such as GTEx) to identify genes involved in the etiology of the phenotype (33). In S-PrediXcan, the predicted expression levels are used to correlate with the phenotype in the gene association test. In our study, the GWAS summary statistics of the antibiotic subgroup were used to perform transcriptome imputation and gene-based association testing by S-PrediXcan using a pre-trained model based on GTEx v7 data (the GTEx-V7_HapMap-2017-11-29.tar.gz file in the PredictDB). The infrastructure described was used to impute gene expression at the MHC region (Chr6: 28477797~33448354, GRCh37) (33). The whole blood and the seven GI tissues were also examined.

## Results

### Characterization of CDI Cohort

Demographics and clinical characteristics of CDI cohorts from Phase I, Phase II, and antibiotic subgroups for CDI GWAS are listed in **Table 1**. CDI cases had older age, higher prevalence of antibiotic and proton pump inhibitor (PPI) use, and inflammatory bowel disease (IBD); all of which were known risk factors for CDI. Other factors such as steroid intake, anti-TNF medication, type 2 diabetes, and transplantation history were only significant in the phase I cohort, reflecting underlying heterogeneity among cohorts.

**Table 1:** Demographics and clinical characteristics of cohorts from phase I, phase II and antibiotics subgroup for CDI GWAS.

	Phase I (11,786)			Phase II (3,518)			Antibiotic subgroup (3,753)		
	Case (946)	Control (10840)	P value	Case (214)	Control (3304)	P value	Case (587)	Control (3166)	P value
**Male Sex, n (%)**	414 (43.8)	4126 (38.1)	0.001	68 (31.8)	1079 (32.7)	0.79	245 (41.7)	1040 (32.8)	3.76E-05
**Index age, mean ± SD**	60.3 ± 17.7	59.0± 19.2	0.041	52.7 ± 18.0	49.0 ± 18.7	0.005	61.0 ± 17.1	55.6 ± 18.9	7.67E-11
**Antibiotics, n (%)**	480 (50.7)	2294 (21.2)	5.58E-94	107 (50)	872 (26.4)	8.14E-14	/	/	/
**Chemotherapy, n (%)**	88 (9.3)	998 (9.2)	0.922	15 (7)	454 (13.7)	0.005	60 (10.2)	535 (16.9)	4.74E-05
**PPI, n (%)**	398 (42.1)	2944 (27.2)	1.68E-22	67 (31.3)	818 (24.8)	0.032	262 (44.6)	1175 (37.1)	0.001
**Steroid, n (%)**	288 (30.6)	2137 (19.7)	2.50E-15	51 (23.8)	691 (20.9)	0.311	196 (33.4)	1027 (32.4)	0.651
**Anti-TNF, n (%)**	16 (1.7)	75 (0.7)	0.001	1 (0.5)	14 (0.4)	0.924	3 (0.5)	15 (0.5)	0.904
**Transplant, n (%)**	52 (5.5)	432 (4.0)	0.023	3 (1.4)	75 (2.3)	0.403	22 (3.7)	127 (4)	0.764
**IBD, n (%)**	81 (8.6)	218 (2.0)	1.03E-34	30 (14.0)	60 (1.8)	6.15E-28	36 (6.1)	59 (1.9)	1.46E-09
**T2DM, n (%)**	370 (39.3)	3685 (34.0)	0.001	42 (19.6)	630 (19.1)	0.84	224 (38.2)	1009 (31.9)	0.003

ANOVA was adopted to test the significance of index age and Chi-square test was adopted to test all other variables. IBD, Inflammatory Bowel Disease; PPI, proton pump inhibitors; TNF, tumor necrosis factor; T2DM, Type 2 Diabetes; HIV, human immunodeficiency virus.

### GWAS and Sensitivity Analyses of CDI in the Antibiotic Subgroup

No variants from the meta-analyses of Phase I and II GWAS reached genome-wide significance (p<5 x10^-8^). The Manhattan and QQ plots for Phase I, Phase II GWAS, and the meta-analyses are displayed in **Supplementary Figure 2**. The top SNVs with p < 5×10^-6^ in the meta-analyses are listed in **Supplementary Table 1**. We queried the Open Targets Genetics and GTEx Portal to evaluate the potential functional impact of these top variants. The variants rs115062572 (OR = 1.74; 95%CI = 1.41-2.08; p=3.508×10^-6^) and rs149917912 (OR = 1.67; 95%CI =1.37-2.00; p=4.017×10^-6^) showed evidence of functional impact on the neighboring genes. These two variants are located in the MHC region and are in partial linkage disequilibrium (LD) in the population of European ancestry (r^2^ = 0.594). The rs115062572 SNV is a significant eQTL for Neurogenic Locus Notch Homolog Protein 4 (*NOTCH4)* in the whole blood (p=1.4x10^-8^) and is a significant splicing quantitative trait loci (sQTL) for MHC Class I Polypeptide-Related Sequence A (*MICA)* in the small intestine (p=4.2x10^-7^) (**Supplementary Figures 3A, B**). The SNV rs149917912 is an eQTL for *MIR6891* (p=6.3x10^-12^) and a sQTL for *HLA-L* in whole blood (p=8.1x10^-18^) (**Supplementary Figures 3C, D**).

**Figure 2:**
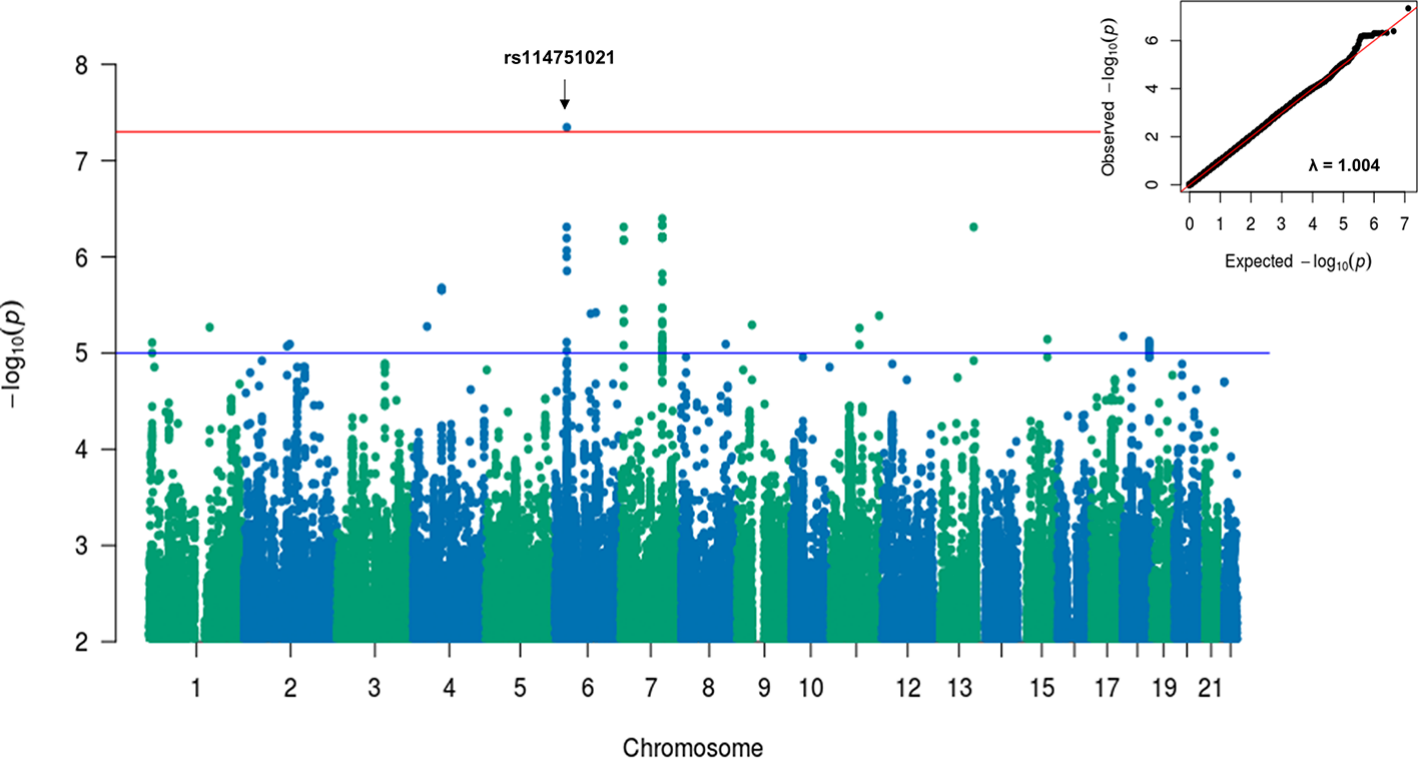
Manhattan and QQ plots for GWAS results associated with CDI in antibiotic treated patients with European ancestry. A linear mixed model regression adjusted for the covariates including sex, cohort, PPI, chemotherapy, T2DM, and Index Age were conducted by BOLT-LMM. Top variants with original p value < 5×10-8 were labeled. The genomic inflation factor, λGC, equals to 1.004, suggesting no evidence for systematic inflation of genome-wide test statistics.

A sensitivity GWAS was performed in a subset of patients with antibiotic treatment to determine whether any of the top associated variants could be enriched in this high-risk cohort. The Manhattan and QQ plot for the sensitivity GWAS are shown in **Figure 2**. Top associated SNVs (p<1×10^-5^) are listed in **Table 2**. The rs114751021 variant in the MHC region is the only genome-wide significant variant associated with CDI (OR = 2.42; 95%CI = 1.84-3.11; p=4.50×10^-8^). The rs114751021 variant is also a significant eQTL for *NOTCH4* in whole blood (p=5.3x10^-14^), and a significant sQTL for *MICA* in adipose tissue (p=1.6x10^-6^) (**Supplementary Figures 3E, F**). This SNV is in partial LD with rs115062572 (r^2^ = 0.593) and rs149917912 (r^2^ = 0.642), both of which demonstrated more significant associations with larger effect-size in the antibiotic subgroup than in the meta-analyses (p = 1.4×10^-6^ and 1.0×10^-6^, respectively). A regional association plot of the rs114751021 shows the top-ranked SNVs in partial LD flank a gene-rich region (**Supplementary Figure 4**). Three other variants showing independent suggestive association at the MHC region are identified after clumping (rs146508039, rs115062572, rs4294047; **Table 2**). The variants rs115062572 (OR=2.14, p=3.0×10^-14^) and rs149917912 (OR=1.53, p=2.0×10^-13^) were significantly associated with IBD or ulcerative colitis according to the UK Biobank (UKB) GWAS results (UKB Neale v2). We further examined the interaction of these SNVs and IBD but did not find significant interactions for these two SNVs in the antibiotic subgroup (p = 0.45 and 0.90, respectively).

**Table 2:** Top CDI-associated variants in the antibiotic subgroup.

**SNV**	**Coordinate**	**A1/A2**	**MAF**	**OR (95%CI)**	**P_BOLT**	**P_SAIGE**	**Gene**
rs114751021	6:31504194	G/A	0.024	2.41 (1.84, 3.09)	4.50E-08	4.35E-07	*SNORD117*
rs6948305	7:109757108	G/C	0.014	2.72 (1.95, 3.67)	3.90E-07	3.36E-06	*-*
rs114995101	13:105278211	G/C	0.020	2.42 (1.8, 3.17)	4.90E-07	1.45E-06	*-*
rs146508039	6:31065037	T/C	0.025	2.24 (1.7, 2.88)	4.90E-07	2.30E-06	*-*
rs78701439	7:9147962	A/G	0.017	2.55 (1.86, 3.39)	4.90E-07	3.99E-06	*-*
rs115062572	6:31862876	T/C	0.022	2.29 (1.7, 2.98)	1.40E-06	5.07E-06	*ZBTB12*
rs140966705	4:75272518	C/T	0.041	1.85 (1.47, 2.28)	2.10E-06	3.03E-06	*-*
rs1419054	6:107084941	A/G	0.115	0.59 (0.43, 0.75)	3.80E-06	6.90E-06	*QRSL1*
rs146471836	6:93817042	T/C	0.026	2.11 (1.59, 2.72)	3.90E-06	6.40E-06	*RP1-23E21.2*
rs183570761	11:127597987	T/C	0.017	2.38 (1.72, 3.17)	4.10E-06	1.75E-05	*-*
rs4142260	9:38389514	T/C	0.466	0.74 (0.65, 0.84)	5.10E-06	2.64E-07	*ALDH1B1*
rs116838950	4:37279460	C/T	0.013	2.65 (1.84, 3.66)	5.30E-06	1.92E-05	*KIAA1239*
rs56040707	1:159521992	G/A	0.027	2.04 (1.55, 2.62)	5.40E-06	9.79E-06	*-*
rs3740779	11:76372052	A/G	0.360	0.73 (0.63, 0.84)	5.50E-06	3.84E-06	*LRRC32*
rs17586705	18:1771063	T/C	0.176	1.42 (1.23, 1.64)	6.80E-06	9.59E-06	*CTD-2015H3.2*
rs118090546	15:77177855	T/G	0.012	2.65 (1.84, 3.69)	7.20E-06	2.63E-05	*SCAPER*
rs73462173	18:69354422	G/A	0.114	0.59 (0.44, 0.76)	7.50E-06	7.98E-06	*-*
rs4294047	6:31101583	A/G	0.103	1.53 (1.28, 1.8)	7.80E-06	1.21E-05	*PSORS1C2*
rs11121431	1:9556558	G/A	0.033	1.92 (1.48, 2.42)	7.90E-06	2.43E-05	*RP13-392I16.1*
rs72675948	8:116048007	T/C	0.027	2.07 (1.56, 2.68)	8.10E-06	2.05E-05	*-*
rs146426342	2:119924607	G/A	0.013	2.54 (1.78, 3.48)	8.20E-06	2.64E-05	*RN7SL468P*
rs115611612	2:112938351	A/T	0.063	1.66 (1.35, 2)	8.40E-06	1.16E-05	*FBLN7*

Variants with p value < 1×10^-5^ in the antibiotic subgroup after clumping are listed. Genomic coordinates are based on hg19 version. Minor allele (A1) is the effect allele. SNVs locating in the MHC region are highlighted in red. MAF, minor allele frequency; P_BOLT, p values from BLOT_LMM; P_SAIGE, p values from SAIGE.

Previously reported CDI-associated SNVS were reviewed. Associations after Bonferroni correction in our study (p<4.5x10^-3^, **Supplementary Table 2**) could not be replicated. The variants rs4073 and rs2227306 in *IL8* showed a nominal significant association in the Phase I cohort (p=0.036 and 0.017), but not in the Phase II cohort (p=0.94 and 0.93 with opposite direction) or the meta-analysis, and the effect size was very small (beta < 0.01). Further, rs2227306 also showed a nominal significant association in the antibiotic subgroup (p=0.026). Another IBD-associated SNV, rs17085007, showed a nominally significant association after meta-analyses, and a small effect size (p=0.039, beta=0.008).

### Colocalization and Transcriptome Imputation-Based Gene-Level Association of the Top Associated SNVs at the MHC Region

**Figure 3:**
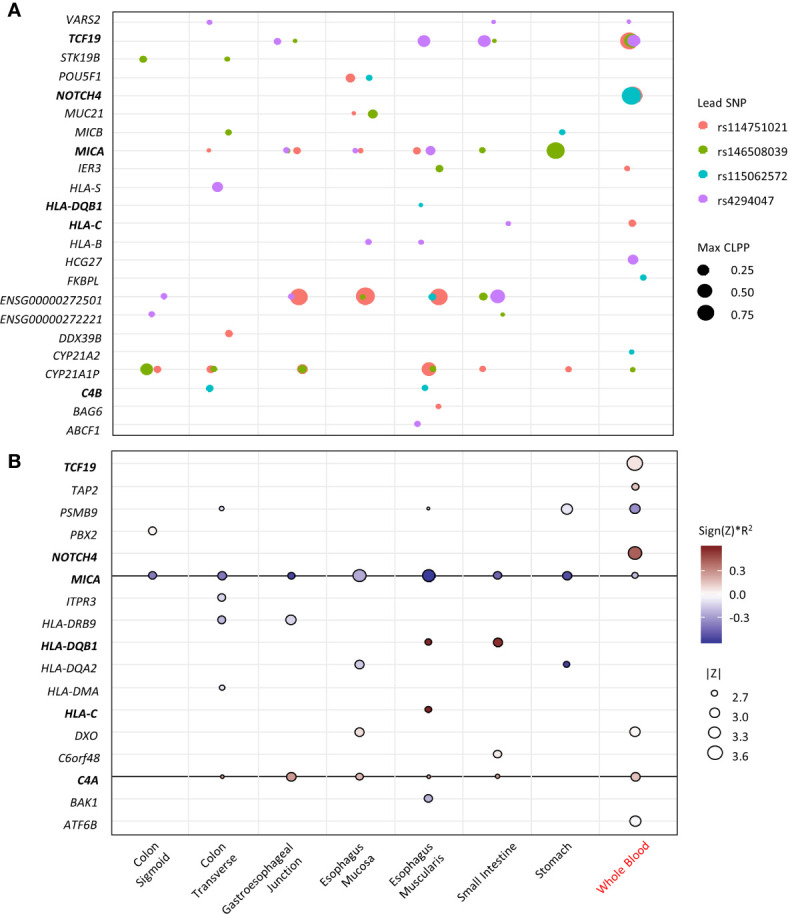
Fine-mapping of the top loci. X-axis represents the tissue types (black indicates GI-tissue). Y-axis represent the genes. Genes that appear in both eCAVIAR and MetaXcan are in blood. **(A)** Colocalization of the 4 lead SNVs at the MHC region. The dot size represents the maximum CLPP values in the corresponding locus and tissues. The color represents different locus for each lead SNV. **(B)** MetaXcan associations of the MHC region with CDI in antibiotic subgroup. The size of the dots represents the significance of the association between predicted expression and the CDI in patients exposed to high-risk antibiotics. Red indicates positive correlation while blue negative. Darker color indicates larger genetic component and consequently more active regulation in the tissue (R^2^ is a model performance measure computed as the correlation squared between observed and predicted expression, cross validated in the training set). Only associations with p<0.01 were shown.

To identify potential causal variant and target genes, colocalization analyses of the four top loci at the MHC region with eQTL data were performed. The variants for each lead SNV that have CLPP >0.01 are listed in **Supplementary Table 3**. The lead SNV is the only variant in each locus that has GWAS p <1x10^-5^ and CLPP >0.01. The target genes and the relevant tissues for each locus are visualized in **Figure 3A**. The genes *TCF19*, *MICA*, *ENSG00000272501*, and *CYP21A1P* are potential target genes for multiple loci in most tissue types. The gene *NOTCH4* is the target gene of two lead SNVs in whole blood, with large posterior probability (CLPP= 0.99 and 0.98). The gene *C4B* is the target gene of rs115062572 in two GI-tissue types. Several HLA genes are also targeted by specific SNV in specific tissues.

The transcriptome imputation-based gene-level association for CDI in the antibiotic subgroup in multiple GI-tract tissues and whole blood was performed. Genes with a nominal significance level (p<0.05) are summarized in **Supplementary Table 4**. Genes with p<0.01 are visualized in **Figure 3B**. Expression in eight genes from whole blood are associated with CDI, of which *NOTCH4* showed the greatest association. The expression of *MICA and C4A* in multiple GI-tract tissues and whole blood are significantly associated with CDI. Expression of *HLA-DRB9, -DQB1, -DQA2, -DMA*, and *-C* in some GI-tissues, but not blood is associated with CDI. *MICA, HLA-DQB1, -DQA2*.

## Discussion

In this study, we applied the eMERGE CDI algorithm to identify CDI cases and controls from a de-identified EHR database and performed GWAS followed by fine-mapping analyses. Although no genome-wide significant associations were identified after meta-analysis, the variants at the MHC region were prioritized with evidence of functional impact on neighborhood genes. The sensitivity GWAS in a subgroup analysis of high-risk patients with antibiotic use prior to CDI showed enhanced associations at the MHC region with the rs114751021 variant reaching genome-wide significance (p=4.50×10^-8^, OR = 2.42[1.84-3.11]). Fine-mapping of the top four lead SNVs at the MHC region by colocalization analyses and transcriptional imputation association test identified genes with potential functional significance in CDI, including *MICA*, *C4A*, *C4B*, and *NOTCH4*, in the GI-tissues and whole blood.

The symptomatic severity of CDI is the result of complex interactions among *C. difficile*, gut microbiota, and host factors. Host risk factors, such as older age, certain comorbidities, and exposure to antibiotics, were observed in this study (**Table 1**). The host immune response is another key determinant of CDI severity. The early immune response to CDI is characterized by the recruitment of neutrophils that eliminate the bacteria through phagocytosis. In addition, they secrete cytokines including IL-1β which contribute to the amplification of the inflammatory response (8). Our findings in this study may shed light on the role of the immune response during CDI. The variants at the MHC region identified in the meta-analyses showed a stronger association with larger effect size in the antibiotic subgroup. Through fine-mapping, we identified four potential target genes: *MICA*, *C4A*, *C4B*, and *NOTCH4*.

The variant *MICA* was identified in most of the GI-tissue types (**Figure 3**). It encodes the highly polymorphic major histocompatibility complex class I chain-related protein A, which serves as one of the ligands for NKG2D, an activating receptor constitutively expressed on natural killer (NK) cells, γδ T cells and other types of killer T cells (34). As with all other NKG2D ligands, *MICA* expression in epithelial cells is induced by stress signals caused by infection (34). For example, *Escherichia coli* increases MICA protein expression levels on the surface of intestinal epithelial cells by stimulating CD55 (35). In our study, a negative association of CDI risk and the *MICA* expression level in all the GI-tissues and blood (**Figure 3B**), was observed, indicating the induction of *MICA* expression could play a critical role in the response to *C. difficile* infection. Failure or altered expression of *MICA* could impose a higher risk for CDI.

We speculate that the recognition of *C. difficile* by epithelial cells induces *MICA* expression, leading to MICA activation of the NKG2D on NK cells and γδ T cells in the epithelium, triggering cell-mediated cytolysis in the presence of proinflammatory cytokines. Studies have found pathogens especially viruses, have evolved mechanisms to inhibit the expression of *MICA* on the infected host cells through masking, internalization, or retention to escape the attack from the host immune system (36). Reduction of *MICA* expression could be an important cause or contributing factor for CDI and restoration of *MICA* expression in the gut could be a treatment strategy for infection. However, whether *C. difficile* possesses a similar mechanism to that of viruses remained to be determined.

The genes *C4A* and *C4B* encode for complement factor C4, a protein belonging to the complement system, which plays an important role in anti-bacterial response (37). Specifically, C4 is cleaved into a small fragment C4_a_ and a large fragment C4_b_, the latter is a key component together with C2a of C3 convertase (C4_b_2_a_) (37). Though the gram-positive cell wall of *C. difficile*, composed of a thick peptidoglycan layer that may be resistant to direct bacterial killing through the formation of a membrane attack complex (MAC), it can be opsonized to attract phagocytes and NK cells (38). The complement fragments C4_b_, C3_b,_ and C1_q_ are important and serve as opsonins. Once adhered to pathogens, they can be recognized by the complement receptor 1 which is expressed on all phagocytes to activate phagocytosis (38). Inmouse models of CDI, C4_b_ is also important to activate C3, which participates in the elimination of translocated bacteria following *C. difficile* disruption of the colonic epithelium (39). These findings highlight the important role of C4 in the severity and outcome of CDI.

*The NOTCH4* gene encodes for NOTCH4, a member of the Notch receptor family. Studies of NOTCH4 in infections are very limited. A study showed that *Notch4*-deficient mice and mice treated with Notch inhibitors were more resistant to *Mycobacterium tuberculosis* infection (40). Notch4 inhibited *M. tuberculosis*–triggered production of proinflammatory cytokines in macrophages through the inhibition of phosphorylation and ubiquitination of TAK1 (40). This is consistent with our finding that upregulated *NOTCH4* is associated with a higher risk for CDI, indicating Notch inhibitors could be potential treatments for CDI.

Epithelial cells produce pro-inflammatory chemokines such as IL8, CXCL1, and CCL2, upon stimulation with *C. difficile* toxins (8). Previous studies have found that variants in *IL8* were associated with CDI, recurrent CDI, or severe CDI using a dominant or a genotypic genetic model (15, 16, 41). However, the sample sizes in previous studies were too small (38, 23, and 18 cases) to achieve sufficient power to detect any associations with CDI. We did not replicate any of these associations in our study (**Supplementary Table 2**).

Although our study represents the largest GWAS for CDI to date, it has several limitations. The *C. difficile* strain information, which can be associated with virulence, was not available for most of the cohort, since presumptive strain type for the NAP-1 strain was only available after mid-April, 2014. Disruption of the gut microbiota was difficult to quantify, even though we included antibiotics and PPIs use–confounders that can interfere with the balance of gut microbiota. Disruption of gut microbiota was not routinely reported for most of the cohort, but as the EHR now contains information on dysbiosis, the future assessment is possible. In addition, detailed information on the antibiotics used was not available in the dataset used, so analysis to further stratify subgroups based on antibiotic exposure was not able to be performed. The use of different antibiotics could have major effects on CDI patients such as the gut microbiota and risk of recurrence. This will be included in the next phase of analysis. A demographic limitation is that the patients in our study are of predominantly European descent and from a single healthcare system. Lastly, replication in a second cohort to validate our findings was not possible as other publicly available cohorts do not have all the data elements needed for the analyses, even though a generalizable phenotyping algorithm was used.

In conclusion, while no genetic variants were identified that met genome-wide significance, several variants within the MHC region had suggestive associations coupled with mechanistic plausibility based on current knowledge of CDI pathogenesis. Fine-mapping identified the genes *MICA*, *C4A/C4B*, and *NOTCH4* to be of potential interest for future studies based on associated evidence of their relevance to the host response to CDI. These results need to be validated in future studies in independent and more diverse cohorts and potential mechanisms require exploration using complementary and orthogonal investigations.

## Data Availability Statement

The datasets presented in this study can be found in online repositories. The names of the repository/repositories and accession number(s) can be found in the article/**Supplementary Material**.

## Author Contributions

ML, VA, JL, and YZ designed the study. YZ and JL performed all analyses and drafted the manuscript. VA and ML supervised the project. DW provided critical input on laboratory data elements and laboratory data validation. AK extracted and de-identified the clinical data from electronic health records. All authors provided critical feedback on the manuscript. All authors contributed to the article and approved the submitted version.

## Funding

This work was supported by Regeneron Genetic Center which provided funds for MyCode consenting, sample collection, genotyping, and genetic data processing. The internal fund to MTML from Geisinger and the external fund from the Defense Threat Reduction Agency to JB-R, RH, and VA (HDTRA1-18-1-0008) supported the data extraction and analyses. The funders had no role in study design, data collection, and interpretation, or the decision to submit the work for publication.

## Conflict of Interest

The authors declare that the research was conducted in the absence of any commercial or financial relationships that could be construed as a potential conflict of interest.
