# Supplementary material for: Variants at the MHC Region Associate With Susceptibility to Clostridioides difficile Infection: A Genome-Wide Association Study Using Comprehensive Electronic Health Records
Source: Front Immunol. 2021 Mar 25;12:638913. doi: 10.3389/fimmu.2021.638913 (PMC8026859; doi:10.3389/fimmu.2021.638913)
Supplement: Supplementary file 1 [file DataSheet_1.docx]

# **Supplementary method**

**Genotyping, imputation and quality control**

The samples were genotyped using Infinium OmniExpress Exome array (Illumina), and GSA-24v1-0 array (Illumina) for Phase I and II, respectively. Genotypes for both cohorts were imputed to HRC.r1-1 EUR reference genome (GRCh37 build) separately using the Michigan Imputation Server, which employed Eagle v2.3 and Minimac4 as the phasing and imputation algorithms. Samples with genotyping rate below 95% were excluded. SNPs with an imputation info score of <0.7, minor allele frequency (MAF) < 1%, and significant deviation from Hardy-Weinberg equilibrium (HWE, p< 10^-4^) were excluded. A pruned set of SNPs was generated from high quality genotyped SNPs (MAF > 0.05, p_hwe_ >0.0001, LD pruned with r^2^ < 0.2) to calculate the relatedness and perform the principal component (PC) analysis (PCA). In total, 2598, 1622 and 216 pairs of individuals with first- or second-degree relatedness were identified in Phase I, II and the antibiotics group, respectively (Supplementary figure A). PCA indicated that the individuals in this study are of European ancestry (Supplementary Figure B). PLINK 1.9 was used for genotypic quality control.

**
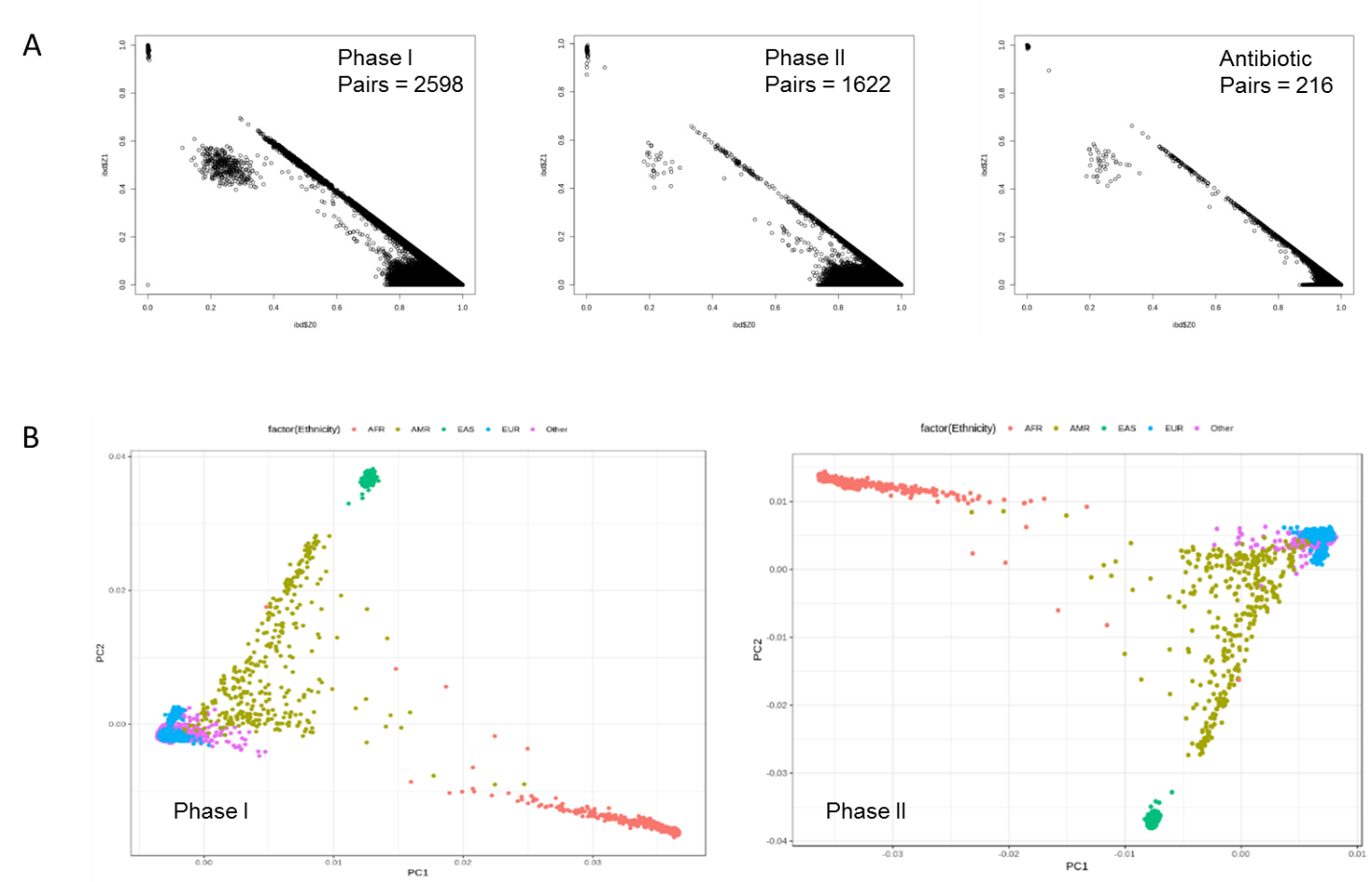
**

# **Supplementary Figure 1: Relatedness and the principal components**

(A) Relatedness plots for Phase I, II and the antibiotics subgroup. Scatter plots of the proportion of loci where the pair shares one allele (Z1) and the proportion of loci where the pair shares zero alleles (Z0) were plotted. No duplicates or monozygotic twins were identified. The number of pairs of 1st or 2nd degree relatedness (PI_HAT > 0.2) is shown in each panel.

(B) Principal component analysis of Phase I and II subjects against 1092 individuals from 1000 Genome Phase 1 data (AFR, n=246; AMR, n=181; ASN, n=286; EUR, n=379). All subject in this study (purple) are closely clustered with samples of the European Ancestry (blue).

**
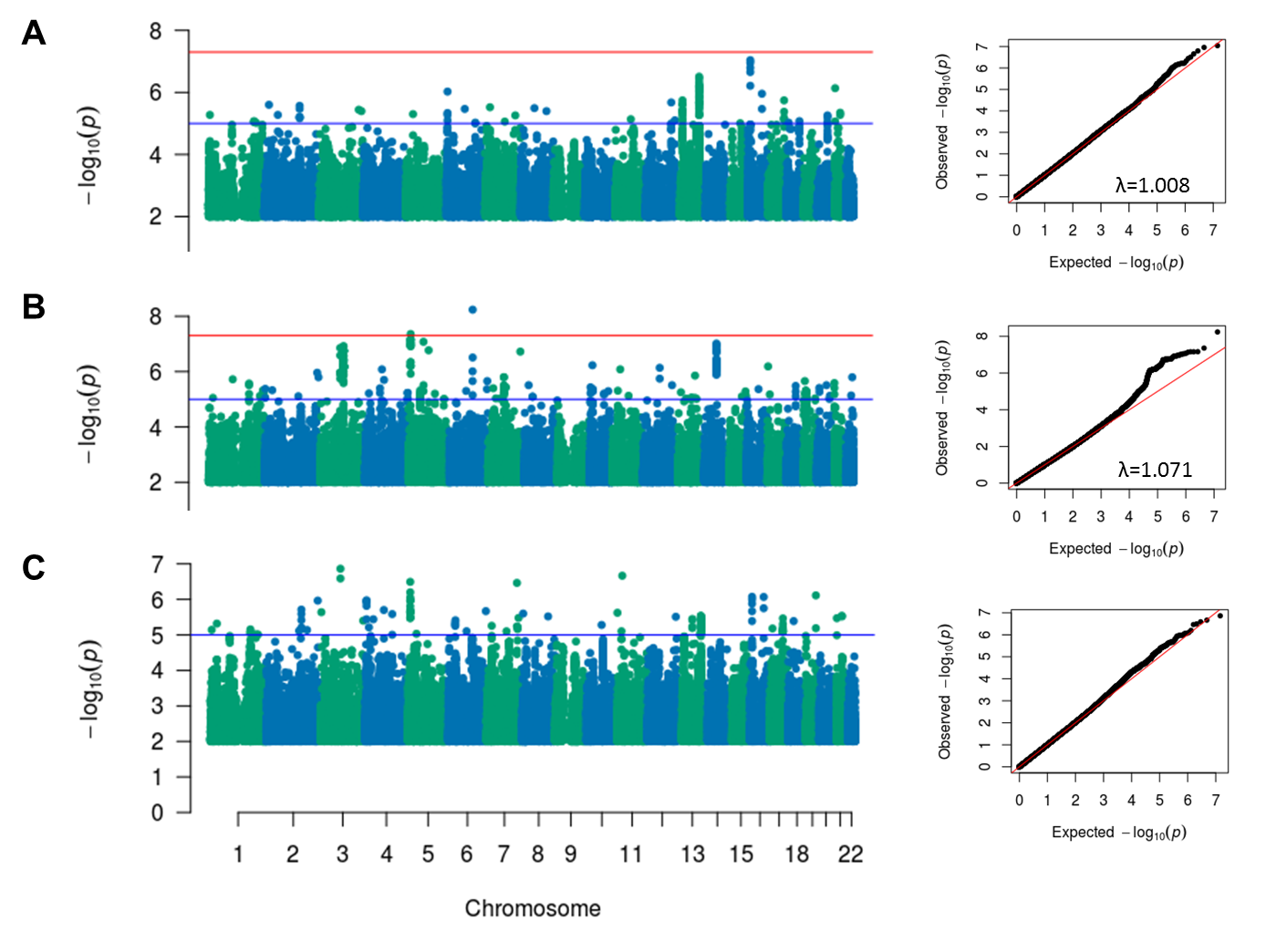
**

# **Supplementary Figure 2. The** **Manhattan and QQ plots for GWAS of Phase I (A), Phase II (B) and the meta-analyses (C).**

A linear mixed model adjusted for the covariates including sex, PPI, chemotherapy, T2DM, and Index Age were conducted by BOLT-LMM.


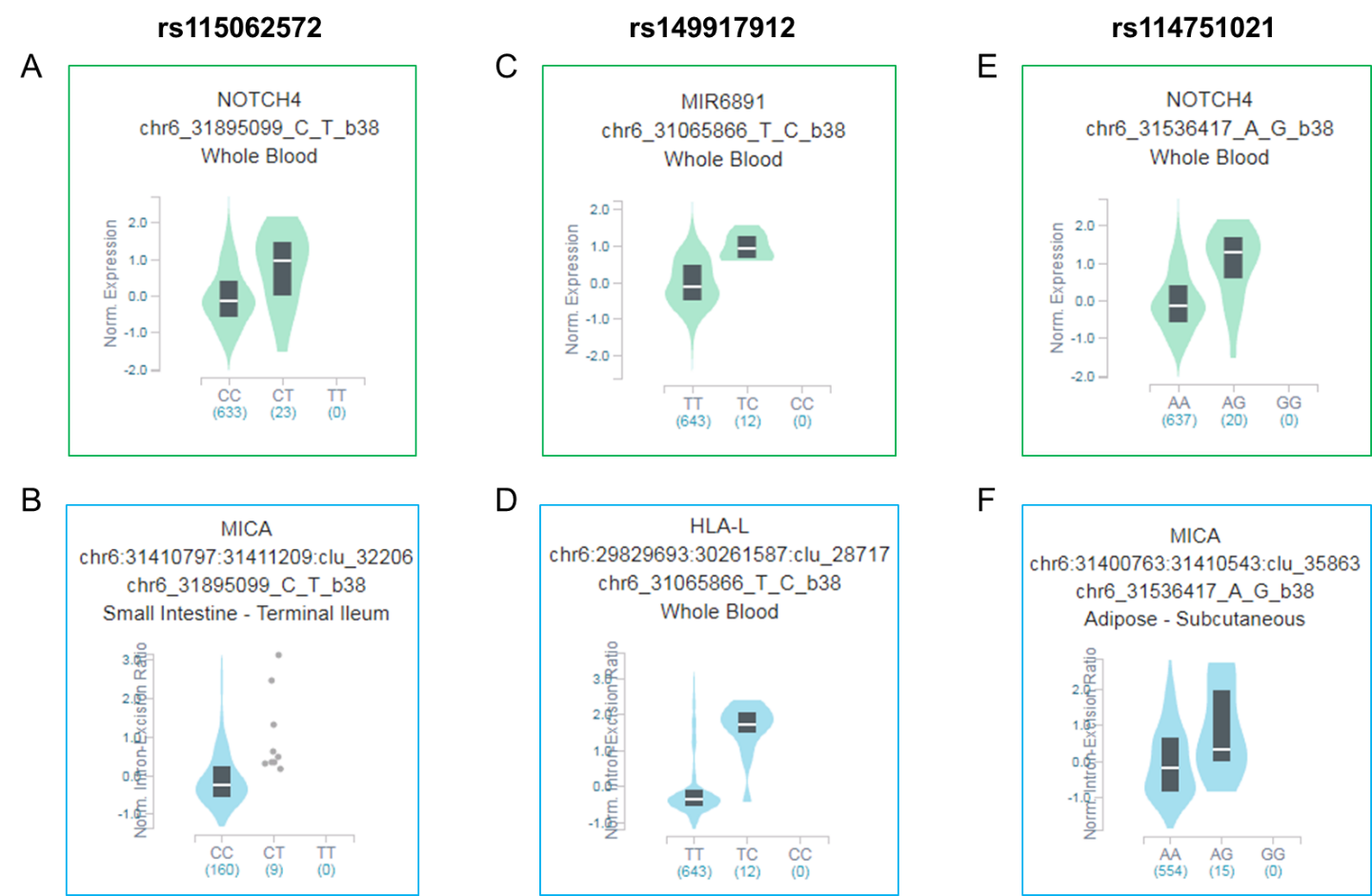


# **Supplementary Figure 3.** **The functional impact of top associated variants at the MHC region from the meta-analyses or sensitivity GWAS of antibiotic subgroup.**

(A) The violin plot of the normalized expression of *NOTCH4* by genotypes of rs115062572 in the whole blood. (B) The violin plot of the normalized intron excision ration of *MICA* by genotypes of rs115062572 in the small intestine. (C) The violin plot of the normalized expression of *MIR6891* by genotypes of rs149917912 in the whole blood. (D) The violin plot of the normalized intron excision ration of *HLA-L* by genotypes of rs115062572 in the whole blood. Data and plots were adopted from GTEx v8 release from GTEx portal. (E) The violin plot of the normalized expression of *NOTCH4* by genotypes of rs11471021 in the whole blood. (F) The violin plot of the normalized intron excision ration of *MICA* by genotypes of rs11471021 in the subcutaneous adipose.

**
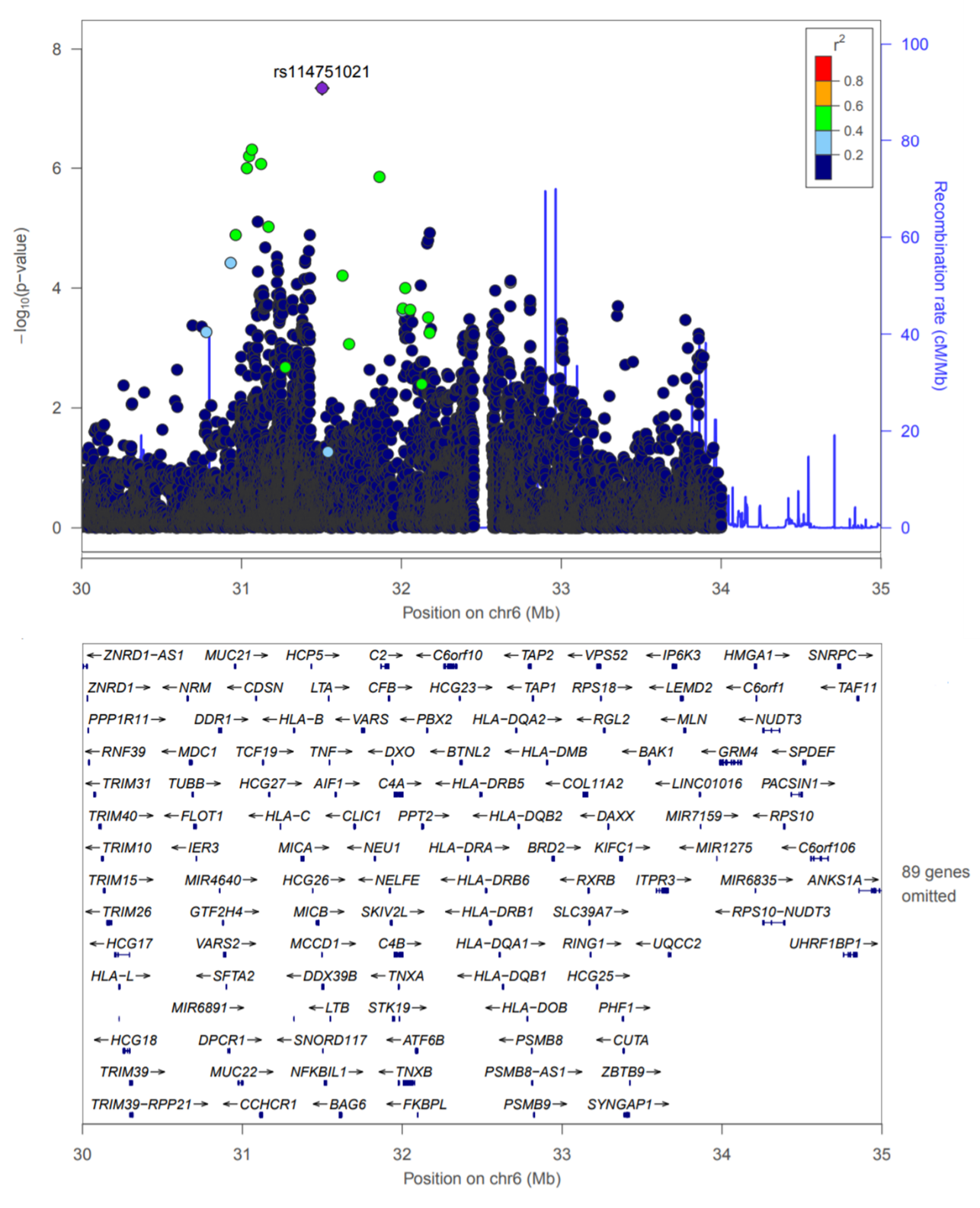
**

# **Supplementary Figure 4. Regional plot for rs114751021 at the MHC region in the subgroup of patients with antibiotics treatment.**

Purple diamond indicated the most significant SNP (rs114751021) at this region. Chromosomal positions and LD were based on hg19/1KG (April 2014) EUR. Colors represent SNPs in LD (r^2^) with the top SNP. These SNPs in partial LD flank a gene-rich region, including lots of immune genes, such as *HLA-C, HLA-DQB1*, *C4A, C4B, C2*, *NOTCH4*. The regional association plot was created by LocusZoom.

# **Supplementary Table 1. Top variants associated with CDI from the meta-analyses of Phase I and II GWAS**

| **SNP** | **Coordinates** | **A1/A2** | **MAF** | **Gene** | **Meta** | | | **Phase I** | | **Phase II** | |
| --- | --- | --- | --- | --- | --- | --- | --- | --- | --- | --- | --- |
|  |  |  |  |  | **OR (95%CI)** | **P** | **#C** | **OR** | **P** | **OR** | **P** |
| rs35294279 | 11:23929633 | C/T | 0.037 | *-* | 1.67 (1.39, 1.92) | 2.16E-07 | 0 | 1.71 | 3.50E-03 | 35.18 | 4.10E-06 |
| rs13181507 | 5:10969297 | A/G | 0.0144 | *CTNND2* | 1.98 (1.58,2.4) | 3.21E-07 | 17 | 1.66 | 1.50E-03 | 4.03 | 2.80E-08 |
| rs117373257 | 7:135178604 | A/G | 0.0133 | *CNOT4/*  *TSPAN32/*  *C11orf21* | 2.02 (1.61,2.47) | 3.44E-07 | 0 | 2.03 | 6.90E-06 | 2.48 | 2.90E-03 |
| rs138769755 | 19:45233897 | T/G | 0.0222 | *RF00285* | 1.76 (1.45,2.1) | 7.72E-07 | 1 | 1.6 | 8.30E-04 | 2.5 | 3.80E-05 |
| rs12924396 | 16:10070285 | G/C | 0.4689 | *GRIN2A* | 0.81 (0.75, 0.88) | 8.46E-07 | 4 | 0.79 | 1.40E-06 | 0.92 | 4.20E-01 |
| rs141932469 | 16:61989131 | T/C | 0.0128 | *CDH8* | 2.03 (1.6,2.48) | 8.54E-07 | 1 | 2.07 | 1.20E-05 | 1.84 | 6.70E-02 |
| rs12641357 | 4:7199132 | T/A | 0.1621 | *SORCS2* | 1.32 (1.18, 1.45) | 1.06E-06 | 4 | 1.33 | 1.00E-04 | 1.6 | 4.00E-03 |
| rs13409177 | 2:157718358 | A/C | 0.0573 | *AC096589.2/*  *AC096589.1* | 1.46 (1.27,1.66) | 1.95E-06 | 2 | 1.45 | 2.70E-05 | 1.34 | 1.80E-01 |
| rs139959052 | 3:3895396 | T/G | 0.0288 | *SUMF1* | 1.73 (1.42,2.07) | 2.30E-06 | 0 | 1.63 | 3.40E-03 | 1.98 | 2.90E-04 |
| rs117600357 | 11:2325192 | A/G | 0.0244 | *-* | 1.69 (1.39,2.01) | 2.39E-06 | 0 | 1.78 | 6.60E-06 | 1.66 | 4.20E-02 |
| rs890001 | 8:4071514 | C/G | 0.0206 | *CSMD1* | 1.77 (1.44,2.12) | 2.51E-06 | 0 | 1.58 | 1.80E-03 | 2.49 | 1.30E-04 |
| rs72672568 | 4:121437815 | C/T | 0.0556 | *AC073475.1* | 1.45 (1.27, 1.67) | 2.60E-06 | 1 | 1.68 | 3.60E-05 | 2.16 | 1.20E-02 |
| rs4772629 | 13:105137529 | T/C | 0.1813 | *-* | 0.79 (0.72, 0.87) | 2.91E-06 | 17 | 1.29 | 1.30E-05 | 1.2 | 1.40E-01 |
| rs62222030 | 21:39363970 | C/T | 0.0746 | *DSCR4/*  *KCNJ6* | 1.41 (1.23, 1.59) | 2.91E-06 | 1 | 1.64 | 9.30E-06 | 1.39 | 1.30E-01 |
| rs80176672 | 8:115044266 | T/A | 0.0122 | *-* | 1.96 (1.54, 2.44) | 3.02E-06 | 0 | 12.95 | 4.80E-06 | 2.14 | 2.30E-01 |
| rs17719655 | 7:142402726 | A/C | 0.0113 | *TRBV26* | 2.02 (1.57,2.5) | 3.33E-06 | 0 | 1.54 | 2.80E-02 | 3.99 | 1.50E-07 |
| rs192418381 | 21:17025154 | C/T | 0.0124 | *-* | 1.96 (1.54, 2.44) | 3.39E-06 | 0 | 173.59 | 1.10E-06 | 1.39 | 5.20E-01 |
| rs73321289 | 17:57326488 | T/C | 0.0121 | *GDPD1* | 1.98 (1.55,2.44) | 3.44E-06 | 10 | 2.03 | 3.90E-05 | 2.18 | 1.00E-02 |
| rs1434351 | 13:105153816 | T/G | 0.3535 | *-* | 1.22 (1.12,1.32) | 3.47E-06 | 8 | 1.28 | 4.70E-07 | 1.07 | 5.30E-01 |
| rs11617974 | 13:105045292 | C/A | 0.3288 | *-* | 1.22 (1.12, 1.33) | 3.53E-06 | 9 | 1.31 | 4.60E-07 | 1.07 | 5.20E-01 |
| rs1982305 | 13:66908399 | G/A | 0.2358 | *PCDH9* | 1.25 (1.14, 1.37) | 3.57E-06 | 3 | 1.24 | 3.20E-04 | 1.41 | 1.10E-02 |
| rs116838950 | 4:37279460 | C/T | 0.0116 | *NWD2* | 2 (1.54, 2.44) | 3.63E-06 | 0 | 6.69 | 4.40E-05 | 247.31 | 1.90E-02 |
| rs115062572 | 6:31862876 | T/C | 0.0203 | *ZBTB12/*  *EHMT2/*  *C2/CYP21A2* | 1.74 (1.41,2.08) | 3.88E-06 | 0 | 1.76 | 5.90E-05 | 1.86 | 1.60E-02 |
| rs57118264 | 18:24298286 | A/C | 0.0179 | *AQP4-AS1* | 1.79 (1.44,2.15) | 4.09E-06 | 0 | 1.65 | 8.50E-04 | 2.27 | 1.90E-03 |
| rs149917912 | 6:31033643 | C/T | 0.0242 | *-* | 1.67 (1.37, 2) | 4.53E-06 | 2 | 2.63 | 3.20E-05 | 2.7 | 3.90E-02 |
| rs867720 | 1:30537232 | C/T | 0.2573 | *-* | 1.23 (1.14, 1.35) | 4.81E-06 | 0 | 1.2 | 1.50E-03 | 1.51 | 1.50E-03 |

Variants with p < 5×10^-6^ in the meta-analyses were clumped based on linkage disequilibrium. Genomic coordinate is based on hg19 version. Minor allele (A1) is the effect allele. Two SNPs, rs115062572 and rs149917912, locating in the MHC region are highlighted in red. #C: number of clumped SNPs. MAF: minor allele frequency.

# **Supplementary Table 2: Review of previously reported CDI-associated variants in our study.**

| **SNP** | **Coordinates** | **EA/**  **RA** | **Gene** | **Published Studies** | | | | | | **Ab** | | **Phase 1** | | **Phase 2** | | **Meta** | | |
| --- | --- | --- | --- | --- | --- | --- | --- | --- | --- | --- | --- | --- | --- | --- | --- | --- | --- | --- |
|  |  |  |  | **OR** | **P** | **Study design** | **Case/Control** | **Remarks** | **Ref.** | **Beta** | **p** | **Beta** | **p** | **Beta** | **p** | **Beta** | **p** | **Direction** |
| rs4073 | 4:74606024 | A/T | *IL8* | 3.3 | 0.014 | Candidate gene, CDI; | 38/73 | Dominant Mode | 2006 | 0.017 | 0.052 | 0.007 | **0.036** | -0.0004 | 0.94 | 0.005 | 0.076 | +- |
|  |  |  |  | 2.7 | 0.043 | Candidate gene, recurrent CDI; | 23/73 | Dominant Mode | 2010 |  |  |  |  |  |  |  |  |  |
| rs2227306 | 4:74607055 | T/C | *IL8* |  |  | Candidate gene; CDI, recurrent CDI and **severe CDI** | 65/65  (18/41 for severe CDI) | Genotypic mode for severe CDI; Inconclusive of risk allele and OR | 2018 | 0.019 | **0.026** | 0.008 | **0.017** | -0.0005 | 0.93 | 0.006 | **0.043** | +- |
| rs2243250 | 5:132009154 | A/G | *IL4* | 5.1 | 1.67E-06 | IBD-loci, CDI in IBD patients | 20/152 | *Calculated | 2014 | 0.002 | 0.87 | -0.002 | 0.69 | -0.009 | 0.26 | -0.004 | 0.35 | -- |
| rs6667605 | 1:2502780 | T/C | *TNFRSF14, MMEL1, PLCH2* | 6 | 0.016 | IBD-loci, CDI in UC patients | 29/290 | The study tested 163 variants. No variants passed Bonferroni correction (p < 3e-4) | 2013 | 0.0005 | 0.95 | 0.005 | 0.14 | 0.006 | 0.28 | 0.005 | 0.07 | ++ |
| rs1363907 | 5:96252803 | A/G | *ERAP2, ERAP1, LNPEP* | 2.96 | 0.03 |  |  |  |  | -0.0016 | 0.85 | -0.001 | 0.75 | -0.001 | 0.84 | -0.001 | 0.71 | -- |
| rs10051722 | 5:130104076 | C/A |  | 4.18 | 0.004 |  |  |  |  | -0.017 | 0.062 | -0.008 | 0.026 | -0.01 | 0.1 | -0.009 | 0.006 | -- |
| rs864745 | 7:28180556 | G/A | *CREB5, JAZF1* | 10.93 | 0.0198 |  |  |  |  | 0.0016 | 0.85 | -0.002 | 0.53 | 0.009 | 0.11 | 0.001 | 0.77 | -+ |
| rs4409764 | 10:101284237 | G/T | *NKX2-3* | 0.42 | 0.029 |  |  |  |  | -0.003 | 0.72 | -0.004 | 0.25 | -0.0004 | 0.95 | -0.003 | 0.31 | -- |
| rs11229555 | 11:58408687 | T/G | *CNTF, LPXN* | 0.32 | 0.017 |  |  |  |  | 0.0108 | 0.28 | -0.001 | 0.81 | 0.002 | 0.78 | -0.0002 | 0.95 | -+ |
| rs17085007 | 13:27531267 | G/A |  | 2.56 | 0.018 |  |  |  |  | 0.0117 | 0.28 | 0.009 | 0.043 | 0.005 | 0.52 | 0.008 | **0.039** | ++ |
| rs941823 | 13:41013977 | T/C |  | 2.55 | 0.017 |  |  |  |  | -0.015 | 0.12 | -0.006 | 0.16 | 0.004 | 0.54 | -0.003 | 0.37 | -+ |
| rs13427342 | 2:171762074 |  | *GORASP2* |  | FDR<  0.01 | GWAS of CDI in Multiple myeloma patients undergoing autologous stem cell transplantation | 57/589 | No Raw P value provided. All variants had MAF <1%. The study had limited power. | 2018 | MAF <1%, Not included in our study | | | | | | | | |
| rs2557792 | 2:171776841 |  |  |  |  |  |  |  |  |  |  |  |  |  |  |  |  |  |
| rs1875964 | 5:174484865 |  | *FLJ16171* |  |  |  |  |  |  |  |  |  |  |  |  |  |  |  |
| rs1875968 | 5:174481014 |  |  |  |  |  |  |  |  |  |  |  |  |  |  |  |  |  |
| rs871234 | 5:174485598 |  |  |  |  |  |  |  |  |  |  |  |  |  |  |  |  |  |
| rs16927456 | 8:62439607 |  | *ASPH* |  |  |  |  |  |  |  |  |  |  |  |  |  |  |  |
| rs16918879 | 8:62414887 |  | *RLBP1L1* |  |  |  |  |  |  |  |  |  |  |  |  |  |  |  |

# **Supplementary Table 3. Variants around each lead SNP that had CLPP>0.01 in the colocalization analyses**

| **Lead SNP** | **Coloc_SNP** | **CLPP** | **Target Gene** | **Tissue** | **GWAS P-value** |
| --- | --- | --- | --- | --- | --- |
| rs146508039 (6:31065037) | 6:31060219 | 0.0105398 | *CCHCR1* | Small Intestine | 5.40E-02 |
|  | 6:31061013 | 0.0105229 | *HLA-B* | Gastroesophageal junction | 4.90E-04 |
|  | 6:31061013 | 0.0240676 | *TCF19* | Small Intestine | 4.90E-04 |
|  | 6:31061013 | 0.0298919 | *ENSG00000272501* | Colon Sigmoid | 4.90E-04 |
|  | 6:31061013 | 0.0148234 | *ENSG00000272501* | Stomach | 4.90E-04 |
|  | **6:31065037** | **0.0308361** | ***CYP21A1P*** | **Colon Transverse** | **4.90E-07** |
|  | **6:31065037** | **0.0891874** | ***CYP21A1P*** | **Gastroesophageal junction** | **4.90E-07** |
|  | **6:31065037** | **0.347296** | ***CYP21A1P*** | **Colon Sigmoid** | **4.90E-07** |
|  | **6:31065037** | **0.0313721** | ***CYP21A1P*** | **Esophageal Muscularis** | **4.90E-07** |
|  | **6:31065037** | **0.0154313** | ***CYP21A1P*** | **Whole Blood** | **4.90E-07** |
|  | **6:31065037** | **0.075291** | ***IER3*** | **Esophageal Muscularis** | **4.90E-07** |
|  | **6:31065037** | **0.0177783** | ***MICA*** | **Gastroesophageal junction** | **4.90E-07** |
|  | **6:31065037** | **0.0250863** | ***MICA*** | **Small Intestine** | **4.90E-07** |
|  | **6:31065037** | **0.901692** | ***MICA*** | **Stomach** | **4.90E-07** |
|  | **6:31065037** | **0.027687** | ***MICB*** | **Colon Transverse** | **4.90E-07** |
|  | **6:31065037** | **0.144281** | ***MUC21*** | **Esophageal Mucosa** | **4.90E-07** |
|  | **6:31065037** | **0.0134886** | ***STK19B*** | **Colon Transverse** | **4.90E-07** |
|  | **6:31065037** | **0.0519388** | ***STK19B*** | **Colon Sigmoid** | **4.90E-07** |
|  | **6:31065037** | **0.0110019** | ***TCF19*** | **Gastroesophageal junction** | **4.90E-07** |
|  | **6:31065037** | **0.526863** | ***TCF19*** | **Whole Blood** | **4.90E-07** |
|  | **6:31065037** | **0.0108166** | ***TCF19*** | **Small Intestine** | **4.90E-07** |
|  | **6:31065037** | **0.0129761** | ***ENSG00000272501*** | **Esophageal Mucosa** | **4.90E-07** |
|  | **6:31065037** | **0.0781398** | ***ENSG00000272501*** | **Small Intestine** | **4.90E-07** |
|  | **6:31065037** | **0.0103388** | ***ENSG00000272221*** | **Small Intestine** | **4.90E-07** |
|  | 6:31065423 | 0.0153941 | *TCF19* | Esophageal Muscularis | 1.40E-03 |
|  | 6:31065423 | 0.0155029 | *ENSG00000272501* | Esophageal Muscularis | 1.40E-03 |
|  | 6:31065423 | 0.0113266 | *ENSG00000272501* | Stomach | 1.40E-03 |
|  | 6:31065423 | 0.0152911 | *ENSG00000272501* | Colon Transverse | 1.40E-03 |
|  | 6:31065423 | 0.0151666 | *ENSG00000272501* | Small Intestine | 1.40E-03 |
|  | 6:31065423 | 0.0148616 | *ENSG00000272501* | Gastroesophageal junction | 1.40E-03 |
|  | 6:31065423 | 0.0155056 | *ENSG00000272501* | Esophageal Mucosa | 1.40E-03 |
|  | 6:31065808 | 0.0100253 | *DDX39B* | Whole Blood | 1.50E-02 |
|  | 6:31066827 | 0.0243811 | *PSORS1C1* | Gastroesophageal junction | 1.10E-02 |
|  | 6:31066827 | 0.0176921 | *ENSG00000271581* | Colon Transverse | 1.10E-02 |
| rs4294047  (6:31101583) | 6:31095942 | 0.0147918 | *HCG27* | Whole Blood | 2.60E-04 |
|  | 6:31095942 | 0.0158035 | *TCF19* | Whole Blood | 2.60E-04 |
|  | 6:31095942 | 0.0170243 | *TCF19* | Small Intestine | 2.60E-04 |
|  | 6:31095942 | 0.0468637 | *ENSG00000272501* | Esophageal Muscularis | 2.60E-04 |
|  | 6:31095942 | 0.0441995 | *ENSG00000272501* | Gastroesophageal junction | 2.60E-04 |
|  | 6:31095942 | 0.046729 | *ENSG00000272501* | Stomach | 2.60E-04 |
|  | 6:31095942 | 0.0103011 | *ENSG00000272501* | Colon Sigmoid | 2.60E-04 |
|  | 6:31095942 | 0.0468425 | *ENSG00000272501* | Esophageal Mucosa | 2.60E-04 |
|  | 6:31095942 | 0.0467257 | *ENSG00000272501* | Colon Transverse | 2.60E-04 |
|  | 6:31097453 | 0.0113887 | *TCF19* | Esophageal Muscularis | 1.00E-03 |
|  | 6:31098134 | 0.0103507 | *CYP21A1P* | Colon Sigmoid | 7.50E-03 |
|  | 6:31098134 | 0.0112858 | *HCG21* | Esophageal Mucosa | 7.50E-03 |
|  | 6:31098134 | 0.012852 | *HCG22* | Esophageal Muscularis | 7.50E-03 |
|  | 6:31098134 | 0.0104348 | *LY6G6C* | Esophageal Muscularis | 7.50E-03 |
|  | 6:31098134 | 0.0146168 | *PSORS1C1* | Stomach | 7.50E-03 |
|  | 6:31098134 | 0.0132247 | *PSORS1C1* | Small Intestine | 7.50E-03 |
|  | 6:31098134 | 0.0149307 | *PSORS1C1* | Esophageal Muscularis | 7.50E-03 |
|  | 6:31098344 | 0.020657 | *HLA-C* | Esophageal Muscularis | 2.90E-04 |
|  | 6:31098344 | 0.0101962 | *HLA-C* | Stomach | 2.90E-04 |
|  | 6:31098344 | 0.137966 | *MICB* | Whole Blood | 2.90E-04 |
|  | 6:31098344 | 0.0192815 | *SNHG32* | Colon Transverse | 2.90E-04 |
|  | 6:31098344 | 0.0107045 | *STK19B* | Esophageal Muscularis | 2.90E-04 |
|  | 6:31098344 | 0.0136588 | *ENSG00000272501* | Colon Sigmoid | 2.90E-04 |
|  | 6:31098344 | 0.117787 | *ENSG00000272501* | Whole Blood | 2.90E-04 |
|  | 6:31100768 | 0.0114931 | *ABCF1* | Esophageal Muscularis | 6.20E-04 |
|  | 6:31100768 | 0.0158341 | *C4A* | Esophageal Mucosa | 6.20E-04 |
|  | 6:31100768 | 0.0191264 | *HLA-S* | Whole Blood | 6.20E-04 |
|  | 6:31100768 | 0.0160824 | *HLA-S* | Colon Transverse | 6.20E-04 |
|  | 6:31101426 | 0.0101864 | *HCG18* | Colon Sigmoid | 3.30E-02 |
|  | **6:31101583** | **0.0350622** | ***ABCF1*** | **Esophageal Muscularis** | **7.70E-06** |
|  | **6:31101583** | **0.17703** | ***HCG27*** | **Whole Blood** | **7.70E-06** |
|  | **6:31101583** | **0.0170177** | ***HLA-B*** | **Esophageal Muscularis** | **7.70E-06** |
|  | **6:31101583** | **0.0392152** | ***HLA-B*** | **Esophageal Mucosa** | **7.70E-06** |
|  | **6:31101583** | **0.0212947** | ***HLA-C*** | **Small Intestine** | **7.70E-06** |
|  | **6:31101583** | **0.176643** | ***HLA-S*** | **Colon Transverse** | **7.70E-06** |
|  | **6:31101583** | **0.0458081** | ***MICA*** | **Gastroesophageal junction** | **7.70E-06** |
|  | **6:31101583** | **0.135348** | ***MICA*** | **Esophageal Muscularis** | **7.70E-06** |
|  | **6:31101583** | **0.0225028** | ***MICA*** | **Esophageal Mucosa** | **7.70E-06** |
|  | **6:31101583** | **0.355869** | ***TCF19*** | **Whole Blood** | **7.70E-06** |
|  | **6:31101583** | **0.332114** | ***TCF19*** | **Esophageal Muscularis** | **7.70E-06** |
|  | **6:31101583** | **0.31656** | ***TCF19*** | **Small Intestine** | **7.70E-06** |
|  | **6:31101583** | **0.0475757** | ***TCF19*** | **Gastroesophageal junction** | **7.70E-06** |
|  | **6:31101583** | **0.0104178** | ***VARS2*** | **Small Intestine** | **7.70E-06** |
|  | **6:31101583** | **0.0186267** | ***VARS2*** | **Colon Transverse** | **7.70E-06** |
|  | **6:31101583** | **0.0111438** | ***VARS2*** | **Whole Blood** | **7.70E-06** |
|  | **6:31101583** | **0.500797** | ***ENSG00000272501*** | **Small Intestine** | **7.70E-06** |
|  | **6:31101583** | **0.0327047** | ***ENSG00000272501*** | **Gastroesophageal junction** | **7.70E-06** |
|  | **6:31101583** | **0.0333931** | ***ENSG00000272501*** | **Colon Sigmoid** | **7.70E-06** |
|  | **6:31101583** | **0.0312129** | ***ENSG00000272221*** | **Colon Sigmoid** | **7.70E-06** |
|  | 6:31101674 | 0.0208519 | *CCHCR1* | Colon Sigmoid | 8.00E-03 |
|  | 6:31101674 | 0.013304 | *HCG21* | Esophageal Mucosa | 8.00E-03 |
|  | 6:31101674 | 0.0105934 | *LY6G6C* | Esophageal Muscularis | 8.00E-03 |
|  | 6:31101674 | 0.0148068 | *PSORS1C1* | Colon Sigmoid | 8.00E-03 |
|  | 6:31101674 | 0.0151661 | *PSORS1C1* | Stomach | 8.00E-03 |
|  | 6:31101674 | 0.0264062 | *PSORS1C1* | Small Intestine | 8.00E-03 |
|  | 6:31101674 | 0.0142693 | *PSORS1C1* | Esophageal Muscularis | 8.00E-03 |
|  | 6:31101674 | 0.026278 | *PSORS1C1* | Esophageal Mucosa | 8.00E-03 |
|  | 6:31101674 | 0.0203805 | *PSORS1C1* | Colon Transverse | 8.00E-03 |
|  | 6:31101778 | 0.0198012 | *ABCF1* | Esophageal Muscularis | 6.70E-04 |
|  | 6:31101778 | 0.0112904 | *C4A* | Esophageal Mucosa | 6.70E-04 |
|  | 6:31101778 | 0.0131836 | *HLA-S* | Colon Transverse | 6.70E-04 |
|  | 6:31101778 | 0.0127206 | *HLA-S* | Whole Blood | 6.70E-04 |
|  | 6:31101917 | 0.0128421 | *CCHCR1* | Colon Sigmoid | 7.80E-03 |
|  | 6:31101917 | 0.0108535 | *LY6G6C* | Esophageal Muscularis | 7.80E-03 |
|  | 6:31101917 | 0.0145854 | *PSORS1C1* | Esophageal Muscularis | 7.80E-03 |
|  | 6:31101917 | 0.0161064 | *PSORS1C1* | Esophageal Mucosa | 7.80E-03 |
|  | 6:31101917 | 0.0207786 | *PSORS1C1* | Colon Transverse | 7.80E-03 |
|  | 6:31101917 | 0.015466 | *PSORS1C1* | Stomach | 7.80E-03 |
|  | 6:31101917 | 0.0209016 | *PSORS1C1* | Colon Sigmoid | 7.80E-03 |
|  | 6:31101917 | 0.0166667 | *TCF19* | Gastroesophageal junction | 7.80E-03 |
|  | 6:31102373 | 0.0185685 | *HCG27* | Whole Blood | 5.20E-05 |
|  | 6:31102373 | 0.0674051 | *HLA-S* | Colon Transverse | 5.20E-05 |
|  | 6:31102373 | 0.0224029 | *MICA* | Gastroesophageal junction | 5.20E-05 |
|  | 6:31102373 | 0.0667351 | *MICA* | Esophageal Muscularis | 5.20E-05 |
|  | 6:31102373 | 0.0709461 | *TCF19* | Esophageal Muscularis | 5.20E-05 |
|  | 6:31102373 | 0.0202988 | *TCF19* | Whole Blood | 5.20E-05 |
|  | 6:31102373 | 0.0136643 | *TCF19* | Small Intestine | 5.20E-05 |
|  | 6:31102373 | 0.0128895 | *TCF19* | Gastroesophageal junction | 5.20E-05 |
|  | 6:31102373 | 0.0110503 | *VARS2* | Colon Transverse | 5.20E-05 |
|  | 6:31106268 | 0.0111713 | *CCHCR1* | Colon Transverse | 9.70E-03 |
|  | 6:31106268 | 0.0107597 | *HLA-C* | Whole Blood | 9.70E-03 |
|  | 6:31106268 | 0.0110885 | *HLA-C* | Colon Transverse | 9.70E-03 |
|  | 6:31106268 | 0.0111752 | *ENSG00000272221* | Esophageal Muscularis | 9.70E-03 |
|  | 6:31106268 | 0.0105357 | *ENSG00000272221* | Colon Transverse | 9.70E-03 |
|  | 6:31106268 | 0.010995 | *ENSG00000272221* | Esophageal Mucosa | 9.70E-03 |
|  | 6:31106268 | 0.0111579 | *ENSG00000272221* | Whole Blood | 9.70E-03 |
|  | 6:31106268 | 0.0111757 | *ENSG00000272221* | Gastroesophageal junction | 9.70E-03 |
|  | 6:31106268 | 0.0108229 | *ENSG00000272221* | Stomach | 9.70E-03 |
| rs114751021 (6:31504194) | **6:31504194** | **0.0196547** | ***BAG6*** | **Esophageal Muscularis** | **4.50E-08** |
|  | **6:31504194** | **0.039303** | ***CYP21A1P*** | **Stomach** | **4.50E-08** |
|  | **6:31504194** | **0.554442** | ***CYP21A1P*** | **Esophageal Muscularis** | **4.50E-08** |
|  | **6:31504194** | **0.186722** | ***CYP21A1P*** | **Gastroesophageal junction** | **4.50E-08** |
|  | **6:31504194** | **0.11198** | ***CYP21A1P*** | **Colon Transverse** | **4.50E-08** |
|  | **6:31504194** | **0.0442475** | ***CYP21A1P*** | **Small Intestine** | **4.50E-08** |
|  | **6:31504194** | **0.0486777** | ***CYP21A1P*** | **Colon Sigmoid** | **4.50E-08** |
|  | **6:31504194** | **0.0618702** | ***DDX39B*** | **Colon Transverse** | **4.50E-08** |
|  | **6:31504194** | **0.0498987** | ***HLA-C*** | **Whole Blood** | **4.50E-08** |
|  | **6:31504194** | **0.0184332** | ***IER3*** | **Whole Blood** | **4.50E-08** |
|  | **6:31504194** | **0.0480239** | ***MICA*** | **Esophageal Muscularis** | **4.50E-08** |
|  | **6:31504194** | **0.0113376** | ***MICA*** | **Colon Transverse** | **4.50E-08** |
|  | **6:31504194** | **0.070111** | ***MICA*** | **Gastroesophageal junction** | **4.50E-08** |
|  | **6:31504194** | **0.0195959** | ***MICA*** | **Esophageal Mucosa** | **4.50E-08** |
|  | **6:31504194** | **0.0105421** | ***MUC21*** | **Esophageal Mucosa** | **4.50E-08** |
|  | **6:31504194** | **0.999048** | ***NOTCH4*** | **Whole Blood** | **4.50E-08** |
|  | **6:31504194** | **0.119899** | ***POU5F1*** | **Esophageal Mucosa** | **4.50E-08** |
|  | **6:31504194** | **0.810935** | ***TCF19*** | **Whole Blood** | **4.50E-08** |
|  | **6:31504194** | **0.959731** | ***ENSG00000272501*** | **Esophageal Mucosa** | **4.50E-08** |
|  | **6:31504194** | **0.814196** | ***ENSG00000272501*** | **Esophageal Muscularis** | **4.50E-08** |
|  | **6:31504194** | **0.834772** | ***ENSG00000272501*** | **Gastroesophageal junction** | **4.50E-08** |
| rs115062572 (6:31862876) | 6:31845985 | 0.0124891 | *HLA-DQB1* | Stomach | 2.50E-02 |
|  | 6:31845985 | 0.0102007 | *HLA-DQB1* | Esophageal Mucosa | 2.50E-02 |
|  | 6:31845985 | 0.0124432 | *HLA-DQB1-AS1* | Stomach | 2.50E-02 |
|  | 6:31845985 | 0.0101075 | *HLA-DQB2* | Whole Blood | 2.50E-02 |
|  | 6:31845985 | 0.0128857 | *HLA-DRB6* | Whole Blood | 2.50E-02 |
|  | 6:31845985 | 0.010302 | *HLA-DRB6* | Stomach | 2.50E-02 |
|  | 6:31845985 | 0.0125226 | *HLA-DRB6* | Colon Transverse | 2.50E-02 |
|  | 6:31845985 | 0.0113371 | *HLA-DRB6* | Colon Sigmoid | 2.50E-02 |
|  | 6:31845985 | 0.0109754 | *ENSG00000272221* | Gastroesophageal junction | 2.50E-02 |
|  | 6:31851234 | 0.0115232 | *MPIG6B* | Esophageal Mucosa | 3.20E-02 |
|  | 6:31851234 | 0.0102249 | *NOTCH4* | Gastroesophageal junction | 3.20E-02 |
|  | 6:31851234 | 0.0110052 | *NOTCH4* | Colon Transverse | 3.20E-02 |
|  | 6:31851234 | 0.01124 | *PSMB9* | Stomach | 3.20E-02 |
|  | 6:31851234 | 0.0114845 | *SLC44A4* | Esophageal Mucosa | 3.20E-02 |
|  | 6:31851234 | 0.0114224 | *ENSG00000272501* | Whole Blood | 3.20E-02 |
|  | **6:31862876** | **0.0505954** | ***C4B*** | **Colon Transverse** | **1.40E-06** |
|  | **6:31862876** | **0.0277838** | ***C4B*** | **Esophageal Muscularis** | **1.40E-06** |
|  | **6:31862876** | **0.0179933** | ***CYP21A2*** | **Whole Blood** | **1.40E-06** |
|  | **6:31862876** | **0.0394671** | ***FKBPL*** | **Whole Blood** | **1.40E-06** |
|  | **6:31862876** | **0.0121836** | ***HLA-DQB1*** | **Esophageal Muscularis** | **1.40E-06** |
|  | **6:31862876** | **0.0296899** | ***MICB*** | **Stomach** | **1.40E-06** |
|  | **6:31862876** | **0.979881** | ***NOTCH4*** | **Whole Blood** | **1.40E-06** |
|  | **6:31862876** | **0.0387535** | ***POU5F1*** | **Esophageal Mucosa** | **1.40E-06** |
|  | **6:31862876** | **0.0710497** | ***ENSG00000272501*** | **Esophageal Muscularis** | **1.40E-06** |
|  | 6:31870326 | 0.0112377 | *C4A* | Esophageal Mucosa | 1.30E-02 |
|  | 6:31870326 | 0.0113835 | *C4A* | Small Intestine | 1.30E-02 |
|  | 6:31870326 | 0.0158907 | *C4A* | Gastroesophageal junction | 1.30E-02 |
|  | 6:31870326 | 0.0113272 | *C4A* | Esophageal Muscularis | 1.30E-02 |
|  | 6:31870326 | 0.0113312 | *C4A* | Colon Transverse | 1.30E-02 |
|  | 6:31870326 | 0.0113272 | *C4A* | Stomach | 1.30E-02 |
|  | 6:31870326 | 0.013528 | *C4A* | Colon Sigmoid | 1.30E-02 |
|  | 6:31870326 | 0.0102495 | *C4B* | Esophageal Mucosa | 1.30E-02 |
|  | 6:31870326 | 0.0103538 | *CCHCR1* | Esophageal Muscularis | 1.30E-02 |
|  | 6:31870326 | 0.0110542 | *CCHCR1* | Colon Transverse | 1.30E-02 |
|  | 6:31870326 | 0.0111899 | *HLA-C* | Esophageal Mucosa | 1.30E-02 |
|  | 6:31870326 | 0.0113199 | *HLA-C* | Esophageal Muscularis | 1.30E-02 |
|  | 6:31870326 | 0.0102988 | *HLA-DQB1* | Esophageal Muscularis | 1.30E-02 |
|  | 6:31870326 | 0.0113079 | *HLA-DQB1* | Colon Transverse | 1.30E-02 |
|  | 6:31870326 | 0.0112797 | *HLA-DQB1* | Esophageal Mucosa | 1.30E-02 |
|  | 6:31870326 | 0.0109918 | *HLA-DQB2* | Esophageal Muscularis | 1.30E-02 |
|  | 6:31870326 | 0.0144381 | *HLA-DRB5* | Colon Sigmoid | 1.30E-02 |
|  | 6:31870326 | 0.0112501 | *HLA-S* | Whole Blood | 1.30E-02 |
|  | 6:31870326 | 0.0113297 | *LY6G5B* | Esophageal Muscularis | 1.30E-02 |
|  | 6:31870326 | 0.0109849 | *MICB* | Stomach | 1.30E-02 |
|  | 6:31870326 | 0.0113277 | *MICB* | Esophageal Mucosa | 1.30E-02 |
|  | 6:31870326 | 0.0100714 | *NOTCH4* | Esophageal Muscularis | 1.30E-02 |
|  | 6:31870326 | 0.0113119 | *PSORS1C1* | Stomach | 1.30E-02 |
|  | 6:31870326 | 0.0103716 | *PSORS1C1* | Colon Sigmoid | 1.30E-02 |
|  | 6:31870326 | 0.0112715 | *PSORS1C1* | Colon Transverse | 1.30E-02 |
|  | 6:31870326 | 0.011324 | *PSORS1C1* | Esophageal Mucosa | 1.30E-02 |
|  | 6:31870326 | 0.0109275 | *PSORS1C1* | Esophageal Muscularis | 1.30E-02 |
|  | 6:31870326 | 0.0113188 | *RNF5* | Stomach | 1.30E-02 |
|  | 6:31870326 | 0.0109652 | *RNF5* | Esophageal Muscularis | 1.30E-02 |
|  | 6:31870326 | 0.0111966 | *RNF5* | Colon Transverse | 1.30E-02 |
|  | 6:31878433 | 0.0116494 | *C4A* | Stomach | 1.20E-02 |
|  | 6:31878433 | 0.0198405 | *C4A* | Whole Blood | 1.20E-02 |
|  | 6:31878433 | 0.0116495 | *C4A* | Esophageal Muscularis | 1.20E-02 |
|  | 6:31878433 | 0.0116515 | *C4A* | Colon Transverse | 1.20E-02 |
|  | 6:31878433 | 0.0117088 | *C4A* | Small Intestine | 1.20E-02 |
|  | 6:31878433 | 0.0117416 | *C4A* | Esophageal Mucosa | 1.20E-02 |
|  | 6:31878433 | 0.0105492 | *C4B* | Esophageal Mucosa | 1.20E-02 |
|  | 6:31878433 | 0.0139381 | *C4B* | Whole Blood | 1.20E-02 |
|  | 6:31878433 | 0.0106497 | *CCHCR1* | Esophageal Muscularis | 1.20E-02 |
|  | 6:31878433 | 0.0113657 | *CCHCR1* | Colon Transverse | 1.20E-02 |
|  | 6:31878433 | 0.0127035 | *CCHCR1* | Whole Blood | 1.20E-02 |
|  | 6:31878433 | 0.0135104 | *CCHCR1* | Gastroesophageal junction | 1.20E-02 |
|  | 6:31878433 | 0.018751 | *CCHCR1* | Colon Sigmoid | 1.20E-02 |
|  | 6:31878433 | 0.0184158 | *CYP21A2* | Whole Blood | 1.20E-02 |
|  | 6:31878433 | 0.0116379 | *HLA-C* | Esophageal Muscularis | 1.20E-02 |
|  | 6:31878433 | 0.0115061 | *HLA-C* | Esophageal Mucosa | 1.20E-02 |
|  | 6:31878433 | 0.01391 | *HLA-DQB1* | Gastroesophageal junction | 1.20E-02 |
|  | 6:31878433 | 0.0116285 | *HLA-DQB1* | Colon Transverse | 1.20E-02 |
|  | 6:31878433 | 0.0115912 | *HLA-DQB1* | Esophageal Mucosa | 1.20E-02 |
|  | 6:31878433 | 0.010591 | *HLA-DQB1* | Esophageal Muscularis | 1.20E-02 |
|  | 6:31878433 | 0.011297 | *HLA-DQB2* | Esophageal Muscularis | 1.20E-02 |
|  | 6:31878433 | 0.018749 | *HLA-DQB2* | Gastroesophageal junction | 1.20E-02 |
|  | 6:31878433 | 0.0116454 | *LY6G5B* | Esophageal Muscularis | 1.20E-02 |
|  | 6:31878433 | 0.011653 | *MICB* | Esophageal Mucosa | 1.20E-02 |
|  | 6:31878433 | 0.0117181 | *MICB* | Gastroesophageal junction | 1.20E-02 |
|  | 6:31878433 | 0.0112951 | *MICB* | Stomach | 1.20E-02 |
|  | 6:31878433 | 0.01036 | *NOTCH4* | Esophageal Muscularis | 1.20E-02 |
|  | 6:31878433 | 0.0116325 | *PSORS1C1* | Stomach | 1.20E-02 |
|  | 6:31878433 | 0.0115923 | *PSORS1C1* | Colon Transverse | 1.20E-02 |
|  | 6:31878433 | 0.0112354 | *PSORS1C1* | Esophageal Muscularis | 1.20E-02 |
|  | 6:31878433 | 0.0116465 | *PSORS1C1* | Esophageal Mucosa | 1.20E-02 |
|  | 6:31878433 | 0.0116295 | *RNF5* | Stomach | 1.20E-02 |
|  | 6:31878433 | 0.0112741 | *RNF5* | Esophageal Muscularis | 1.20E-02 |
|  | 6:31878433 | 0.0115188 | *RNF5* | Colon Transverse | 1.20E-02 |
|  | 6:31878433 | 0.020527 | *STK19B* | Colon Sigmoid | 1.20E-02 |
|  | 6:31879740 | 0.0270327 | *STK19B* | Stomach | 3.40E-03 |
|  | 6:31879740 | 0.0453048 | *STK19B* | Gastroesophageal junction | 3.40E-03 |
|  | 6:31881309 | 0.0117735 | *BAG6* | Esophageal Muscularis | 1.10E-02 |
|  | 6:31881309 | 0.0212218 | *BTNL2* | Small Intestine | 1.10E-02 |
|  | 6:31881309 | 0.0252321 | *CYP21A1P* | Colon Transverse | 1.10E-02 |
|  | 6:31881309 | 0.0143232 | *CYP21A1P* | Stomach | 1.10E-02 |
|  | 6:31881309 | 0.0205982 | *CYP21A1P* | Colon Sigmoid | 1.10E-02 |
|  | 6:31881309 | 0.025231 | *CYP21A1P* | Esophageal Muscularis | 1.10E-02 |
|  | 6:31881309 | 0.0202366 | *CYP21A1P* | Whole Blood | 1.10E-02 |
|  | 6:31881309 | 0.0252134 | *CYP21A1P* | Gastroesophageal junction | 1.10E-02 |
|  | 6:31881309 | 0.0252187 | *CYP21A1P* | Esophageal Mucosa | 1.10E-02 |
|  | 6:31881309 | 0.0166881 | *GPANK1* | Gastroesophageal junction | 1.10E-02 |
|  | 6:31881309 | 0.0168335 | *HLA-C* | Colon Sigmoid | 1.10E-02 |
|  | 6:31881309 | 0.0193614 | *HLA-C* | Colon Transverse | 1.10E-02 |
|  | 6:31881309 | 0.013692 | *HLA-DQA2* | Whole Blood | 1.10E-02 |
|  | 6:31881309 | 0.0138531 | *HLA-DQA2* | Colon Transverse | 1.10E-02 |
|  | 6:31881309 | 0.0110459 | *HLA-DQA2* | Esophageal Mucosa | 1.10E-02 |
|  | 6:31881309 | 0.0201681 | *HLA-DQA2* | Gastroesophageal junction | 1.10E-02 |
|  | 6:31881309 | 0.0161019 | *HLA-DRB1* | Whole Blood | 1.10E-02 |
|  | 6:31881309 | 0.0222142 | *HLA-DRB5* | Gastroesophageal junction | 1.10E-02 |
|  | 6:31881309 | 0.0175262 | *HLA-DRB6* | Gastroesophageal junction | 1.10E-02 |
|  | 6:31881309 | 0.0225947 | *LY6G6C* | Esophageal Mucosa | 1.10E-02 |
|  | 6:31881309 | 0.0153552 | *MUC22* | Esophageal Mucosa | 1.10E-02 |

# **Supplementary Table 4. MetaXcan associations for the MHC-region in the antibiotic subgroup.**

| **Gene** | **Gene name** | **zscore** | **Effect size** | **P value** | **Tissue** |
| --- | --- | --- | --- | --- | --- |
| ENSG00000137310.7 | *TCF19* | 3.833 | 0.0995 | 1.27E-04 | Whole blood |
| ENSG00000204301.5 | *NOTCH4* | 3.445 | 0.0314 | 5.71E-04 |  |
| ENSG00000213676.6 | *ATF6B* | -3.069 | -0.1329 | 2.15E-03 |  |
| ENSG00000240065.3 | *PSMB9* | -3.063 | -0.0348 | 2.19E-03 |  |
| ENSG00000204348.5 | *DXO* | 3.0487 | 0.3397 | 2.30E-03 |  |
| ENSG00000244731.3 | *C4A* | 2.897 | 0.0507 | 3.77E-03 |  |
| ENSG00000204267.9 | *TAP2* | 2.778 | 0.04357 | 5.47E-03 |  |
| ENSG00000204520.8 | *MICA* | -2.714 | -0.03797 | 6.65E-03 |  |
| ENSG00000228432.1 | *DHFRP2* | 2.530 | 0.1452 | 1.14E-02 |  |
| ENSG00000096433.6 | *ITPR3* | 2.463 | 2.2467 | 1.38E-02 |  |
| ENSG00000179344.12 | *HLA-DQB1* | 2.411 | 0.05443 | 1.59E-02 |  |
| ENSG00000272221.1 | *XXbac-BPG181B23.7* | -2.405 | -0.02344 | 1.62E-02 |  |
| ENSG00000137411.12 | *VARS2* | 2.393 | 0.02007 | 1.67E-02 |  |
| ENSG00000237541.3 | *HLA-DQA2* | -2.359 | -0.02332 | 1.83E-02 |  |
| ENSG00000232629.4 | *HLA-DQB2* | -2.256 | -0.03023 | 2.41E-02 |  |
| ENSG00000196301.3 | *HLA-DRB9* | -2.130 | -0.04742 | 3.32E-02 |  |
| ENSG00000166278.10 | *C2* | -2.126 | -0.05194 | 3.35E-02 |  |
| ENSG00000204305.9 | *AGER* | -2.120 | -0.08373 | 3.40E-02 |  |
| ENSG00000224389.4 | *C4B* | -2.0629 | -0.03049 | 3.91E-02 |  |
| ENSG00000204482.6 | *LST1* | -1.999 | -0.05525 | 4.56E-02 |  |
| ENSG00000271581.1 | *XXbac-BPG248L24.12* | -1.963 | -0.03338 | 4.96E-02 |  |
| ENSG00000240065.3 | *PSMB9* | -3.091 | -0.10542 | 2.00E-03 | Stomach |
| ENSG00000204520.8 | *MICA* | -2.963 | -0.03317 | 3.05E-03 |  |
| ENSG00000237541.3 | *HLA-DQA2* | -2.733 | -0.04483 | 6.28E-03 |  |
| ENSG00000196735.7 | *HLA-DQA1* | 2.551 | 0.03580 | 1.07E-02 |  |
| ENSG00000234745.5 | *HLA-B* | -2.523 | -0.05437 | 1.16E-02 |  |
| ENSG00000179344.12 | *HLA-DQB1* | 2.505 | 0.04276 | 1.22E-02 |  |
| ENSG00000244731.3 | *C4A* | 2.491 | 0.03528 | 1.27E-02 |  |
| ENSG00000204308.6 | *RNF5* | 2.417 | 0.03737 | 1.56E-02 |  |
| ENSG00000232629.4 | *HLA-DQB2* | -2.350 | -0.02577 | 1.88E-02 |  |
| ENSG00000204525.10 | *HLA-C* | 2.334 | 0.03617 | 1.96E-02 |  |
| ENSG00000204444.6 | *APOM* | 2.144 | 0.1768 | 3.20E-02 |  |
| ENSG00000137411.12 | *VARS2* | 2.115 | 0.02246 | 3.44E-02 |  |
| ENSG00000179344.12 | *HLA-DQB1* | 2.922 | 0.06221 | 3.48E-03 | Small Intestine |
| ENSG00000204387.8 | *C6orf48* | 2.876 | 0.0637 | 4.03E-03 |  |
| ENSG00000204520.8 | *MICA* | -2.807 | -0.02412 | 5.00E-03 |  |
| ENSG00000244731.3 | *C4A* | 2.649 | 0.03582 | 8.07E-03 |  |
| ENSG00000240065.3 | *PSMB9* | -2.437 | -0.0433 | 1.48E-02 |  |
| ENSG00000204544.5 | *MUC21* | -2.272 | -0.1368 | 2.31E-02 |  |
| ENSG00000272221.1 | *XXbac-BPG181B23.7* | -2.141 | -0.02338 | 3.23E-02 |  |
| ENSG00000137411.12 | *VARS2* | 2.089 | 0.01832 | 3.67E-02 |  |
| ENSG00000241106.2 | *HLA-DOB* | -2.033 | -0.05056 | 4.20E-02 |  |
| ENSG00000237541.3 | *HLA-DQA2* | -1.965 | -0.01965 | 4.94E-02 |  |
| ENSG00000204520.8 | *MICA* | -3.304 | -0.03359 | 9.55E-04 | Esophagus Muscularis |
| ENSG00000030110.8 | *BAK1* | -2.868 | -0.2673 | 4.13E-03 |  |
| ENSG00000204525.10 | *HLA-C* | 2.740 | 0.03635 | 6.14E-03 |  |
| ENSG00000179344.12 | *HLA-DQB1* | 2.733 | 0.0616 | 6.27E-03 |  |
| ENSG00000244731.3 | *C4A* | 2.615 | 0.03032 | 8.93E-03 |  |
| ENSG00000240065.3 | *PSMB9* | -2.607 | -0.06145 | 9.13E-03 |  |
| ENSG00000204482.6 | *LST1* | -2.508 | -0.07988 | 1.21E-02 |  |
| ENSG00000204472.8 | *AIF1* | -2.399 | -0.1161 | 1.65E-02 |  |
| ENSG00000204308.6 | *RNF5* | 2.358 | 0.04105 | 1.84E-02 |  |
| ENSG00000204348.5 | *DXO* | 2.320 | 0.05271 | 2.03E-02 |  |
| ENSG00000232629.4 | *HLA-DQB2* | -2.234 | -0.03088 | 2.55E-02 |  |
| ENSG00000237541.3 | *HLA-DQA2* | -2.211 | -0.03363 | 2.70E-02 |  |
| ENSG00000204301.5 | *NOTCH4* | 2.195 | 0.03225 | 2.82E-02 |  |
| ENSG00000137310.7 | *TCF19* | 2.050 | 0.03794 | 4.03E-02 |  |
| ENSG00000096433.6 | *ITPR3* | -2.048 | -0.2108 | 4.05E-02 |  |
| ENSG00000196301.3 | *HLA-DRB9* | -2.029 | -0.04661 | 4.24E-02 |  |
| ENSG00000204257.10 | *HLA-DMA* | -2.024 | -0.03394 | 4.30E-02 |  |
| ENSG00000204469.8 | *PRRC2A* | -1.996 | -0.0702 | 4.60E-02 |  |
| ENSG00000204520.8 | *MICA* | -3.316 | -0.03945 | 9.13E-04 | Esophagus Mucosa |
| ENSG00000237541.3 | *HLA-DQA2* | -2.937 | -0.04396 | 3.31E-03 |  |
| ENSG00000204348.5 | *DXO* | 2.920 | 0.05555 | 3.50E-03 |  |
| ENSG00000244731.3 | *C4A* | 2.768 | 0.03738 | 5.64E-03 |  |
| ENSG00000204301.5 | *NOTCH4* | 2.429 | 0.02582 | 1.51E-02 |  |
| ENSG00000234745.5 | *HLA-B* | -2.275 | -0.03500 | 2.29E-02 |  |
| ENSG00000272221.1 | *XXbac-BPG181B23.7* | -2.219 | -0.02250 | 2.65E-02 |  |
| ENSG00000204427.7 | *ABHD16A* | -2.093 | -0.0436 | 3.63E-02 |  |
| ENSG00000137310.7 | *TCF19* | -2.076 | -0.02129 | 3.79E-02 |  |
| ENSG00000137411.12 | *VARS2* | 2.051 | 0.02516 | 4.03E-02 |  |
| ENSG00000196301.3 | *HLA-DRB9* | -3.009 | -0.07868 | 2.62E-03 | Esophagus Gastroesophageal  Junction |
| ENSG00000244731.3 | *C4A* | 2.883 | 0.05107 | 3.94E-03 |  |
| ENSG00000204520.8 | *MICA* | -2.786 | -0.0348 | 5.33E-03 |  |
| ENSG00000240065.3 | *PSMB9* | -2.574 | -0.05935 | 1.01E-02 |  |
| ENSG00000237541.3 | *HLA-DQA2* | -2.525 | -0.03335 | 1.16E-02 |  |
| ENSG00000232629.4 | *HLA-DQB2* | -2.489 | -0.04630 | 1.28E-02 |  |
| ENSG00000204257.10 | *HLA-DMA* | -2.480 | -0.08021 | 1.31E-02 |  |
| ENSG00000179344.12 | *HLA-DQB1* | 2.441 | 0.07664 | 1.47E-02 |  |
| ENSG00000137411.12 | *VARS2* | 2.307 | 0.02478 | 2.11E-02 |  |
| ENSG00000204525.10 | *HLA-C* | 2.255 | 0.03482 | 2.44E-02 |  |
| ENSG00000204267.9 | *TAP2* | -2.240 | -0.05233 | 2.51E-02 |  |
| ENSG00000271581.1 | *XXbac-BPG248L24.12* | -2.158 | -0.05136 | 3.10E-02 |  |
| ENSG00000204536.9 | *CCHCR1* | 2.002 | 0.02908 | 4.53E-02 |  |
| ENSG00000234745.5 | *HLA-B* | -1.962 | -0.02372 | 4.98E-02 |  |
| ENSG00000204520.8 | *MICA* | -2.824 | -0.03117 | 4.74E-03 | Colon Sigmoid |
| ENSG00000204304.7 | *PBX2* | 2.812 | 0.19174 | 4.92E-03 |  |
| ENSG00000240065.3 | *PSMB9* | -2.361 | -0.04405 | 1.82E-02 |  |
| ENSG00000244731.3 | *C4A* | 2.346 | 0.03008 | 1.90E-02 |  |
| ENSG00000179344.12 | *HLA-DQB1* | 2.333 | 0.04580 | 1.96E-02 |  |
| ENSG00000137312.10 | *FLOT1* | -2.220 | -0.03335 | 2.64E-02 |  |
| ENSG00000232629.4 | *HLA-DQB2* | -2.081 | -0.03250 | 3.74E-02 |  |
| ENSG00000137411.12 | *VARS2* | 1.985 | 0.02230 | 4.72E-02 |  |
| ENSG00000204525.10 | *HLA-C* | 1.982 | 0.02580 | 4.74E-02 |  |
| ENSG00000204520.8 | *MICA* | -2.947 | -0.03571 | 3.21E-03 | Colon Transverse |
| ENSG00000096433.6 | *ITPR3* | -2.878 | -0.11780 | 4.00E-03 |  |
| ENSG00000196301.3 | *HLA-DRB9* | -2.819 | -0.04712 | 4.82E-03 |  |
| ENSG00000204257.10 | *HLA-DMA* | -2.674 | -0.08206 | 7.49E-03 |  |
| ENSG00000240065.3 | *PSMB9* | -2.630 | -0.08670 | 8.53E-03 |  |
| ENSG00000244731.3 | *C4A* | 2.619 | 0.03941 | 8.81E-03 |  |
| ENSG00000244731.3 | *C4A* | 2.619 | 0.03941 | 8.81E-03 |  |
| ENSG00000137411.12 | *VARS2* | 2.295 | 0.02015 | 2.18E-02 |  |
| ENSG00000237541.3 | *HLA-DQA2* | -2.266 | -0.02845 | 2.34E-02 |  |
| ENSG00000179344.12 | *HLA-DQB1* | 2.237 | 0.04629 | 2.53E-02 |  |
| ENSG00000229391.3 | *HLA-DRB6* | -2.207 | -0.02126 | 2.73E-02 |  |
| ENSG00000204308.6 | *RNF5* | 2.131 | 0.04453 | 3.31E-02 |  |
| ENSG00000232629.4 | *HLA-DQB2* | -1.981 | -0.02788 | 4.76E-02 |  |
| ENSG00000272221.1 | *XXbac-BPG181B23.7* | -1.968 | -0.01644 | 4.91E-02 |  |
